# Development of CuMn_x_O_y_ (x = 2, and y = 4)-GO heterostructure for the synthesis of pyranoquinoline derivatives

**DOI:** 10.1038/s41598-023-36529-y

**Published:** 2023-06-21

**Authors:** Ayda Farajollahi, Nader Noroozi Pesyan, Ahmad Poursattar Marjani, Hassan Alamgholiloo

**Affiliations:** Department of Organic Chemistry, Faculty of Chemistry, Urmia University, Urmia, Iran

**Keywords:** Catalysis, Green chemistry, Organic chemistry

## Abstract

The pyranoquinoline derivatives are synthetically important due to their biological properties. In this research, these derivatives were produced through an environmentally friendly method. This method includes the use of CuMn_x_O_y_ (x = 2, and y = 4)-GO as a nanocatalyst, which is easy to produce, has excellent performance, cost-effectiveness, and recyclability among its features, and also the use of water as a green solvent. Pyranoquinolines through the one-pot, the multi-component reaction between different derivatives of aryl glyoxal, ethyl cyanoacetate, and 4-hydroxyquinoline-2(1*H*)-one were synthesized using nanocatalyst, K_2_CO_3,_ and H_2_O. Also, the structure of the CuMn_x_O_y_-GO nanocatalyst was evaluated and confirmed via different analyses. The distinguishing features of this work compared to previous works are easy workup, recyclability of nanocatalyst, facile synthesis process, and provide high yields of products.

## Introduction

With the increase in population and the emergence of various diseases, the need for drugs has increased, especially drugs that have heterocyclic compounds in their structure. Multicomponent reactions are one of the most important and effective reactions in the field of synthesis of heterocyclic compounds in chemistry, which makes the reaction easy and fast without the need to separate the intermediates^1,2^. The pyranoquinoline derivatives are an important group of heterocyclic compounds in the field of organic chemistry due to pharmaceutical and biological properties such as antimalarial^3^, anticancer^4^, antimicrobial^5^, etc. Many studies have offered various methods for the synthesis of pyranoquinoline derivatives, such as the use of microwave radiation^6^, ultrasonic radiation^7^, room temperature^8^, nanocatalysts^9^, Hack reaction^10^, and Diels–Alder reaction^11^. Therefore, pyranoquinolines were synthesized in the presence of nanocatalysts in a pure form with high efficiency^12^. Moreover, the choice of solvent is important, and in its selection, issues related to cost, safety, and environmental pollution should be considered. In recent years, the use of green solvents has been developed because they are environmentally friendly^13^.

Recently, the use of graphene-based materials in various research fields has attracted the attention of researchers^14,15^. These nanostructures have many applications in the field of drug production, fuel cells, water purification, and many organic reactions due to their features such as high conductivity, high activity, easy separation, and recycling^16–19^.

**Figure 1:**
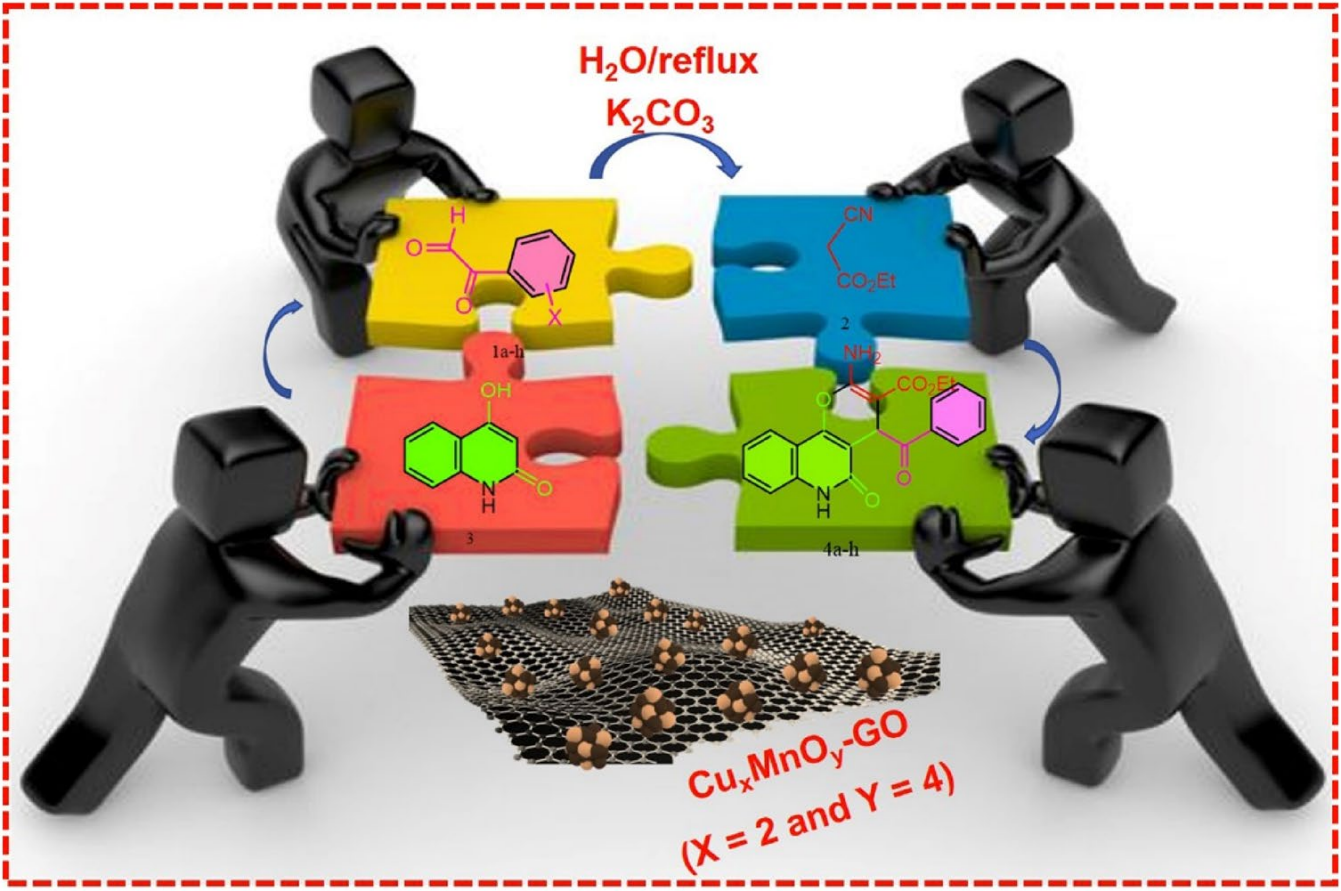
Synthesis of pyranoquinolines derivatives with CuMn_x_O_y_-GO as a heterostructure.

In this study, the CuMn_x_O_y_-GO heterostructure was generated through a new method for the development of pyranoquinoline compounds (Fig. 1). The evaluation of the structure of the nanostructure through different analyses indicated that it was prepared correctly. The presence of copper and manganese nanoparticles on the GO sheets has increased the activity of the nanocatalyst. Furthermore, H_2_O as a suitable solvent for the reaction due to its cheapness, availability, green solvent, and non-toxicity was used for the synthesis of CuMn_x_O_y_-GO nanocatalyst and pyranoquinolines. Pyranoquinolines under different conditions and in the presence of different catalysts have been presented by our research group in many reports^20–24^. But in this work, the CuMn_x_O_y_-GO has features such as easy preparation, high performance, and recyclability and causes the synthesis of pyranoquinolines in high yields.

## Experimental

### Materials

Aryl glyoxal, ethyl cyanoacetate, 4-hydroxyquinolin-2(1*H*)-one, Mn(OAc)_2_, K_2_CO_3_, KMnO_4_, Cu(NO_3_)_2_, ethanol (EtOH), GO (graphene oxide), ethyl acetate (EtOAc), Hexane, and Methanol (MeOH) were obtained from Aldrich and Merck companies.

### Preparation of CuMn_x_O_y_ (x = 2, and y = 4)

CuMn_x_O_y_ (x = 2, and y = 4) nanocatalyst was produced according to the presentation of Niagi^25^. To prepare it, solutions with specific amounts of Mn(CH_3_COO)_2_·4H_2_O (11.05 g/25 mL H_2_O), Cu(NO_3_)_2_·5H_2_O (1.5 g/25 mL H_2_O), and KMnO_4_ (4.73 g/25 mL H_2_O) were prepared. Then, Mn(CH_3_COO)_2_·4H_2_O solution was added to the Cu(NO_3_)_2_·5H_2_O solution. Next, this solution was added dropwise to the KMnO_4_ solution. This mixture was reacted under stirring conditions for 24 h. After this time, the reaction mixture was filtered, rinsed several times with distilled water, and dried at 120 °C. Finally, the product was calcined at 200 °C for 3 h.

### Preparation of CuMn_x_O_y_ (x = 2, and y = 4)-GO nanocatalyst

GO was produced according to standard Hummer’s procedure and then it was delaminated under sonication in the existence of polyethylene glycol )PEG) as a stabilizer^17,18^ (Support Information in Text S1). Firstly, GO (50 mg) and CuMn_x_O_y_ (50 mg) was added in two different flasks in 25 mL of deionized water and placed under ultrasonic conditions for 1.5 h. Then, they were added to a 100 mL flask and subjected to ultrasonication for 50 min. Next, the reaction mixture was placed under stirrer conditions for 24 h for better placement of CuMn_x_O_y_ nanoparticles between GO sheets. After that, the resulting mixture was filtered and rinsed with water, and dried at 120 °C. Lastly, the CuMn_x_O_y_-GO nanoccatalyst was obtained.

### Preparation of ethyl 2-amino-4-benzoyl-5-oxo-5,6-dihydro-4*H*-pyrano[3,2-*c*]quinoline-3-carboxylates

In a reaction container, ethyl cyanoacetate (0.1 mmol, 10 mg), aryl glyoxal (0.1 mmol, 10 mg), and 4-hydroxyquinolin-2(1*H*)-one (0.1 mmol, 16 mg) has been reacted under reflux conditions in the presence of nanocatalyst (CuMn_x_O_y_-GO, 20 mg), K_2_CO_3_ (0.3 mmol, 40 mg) and H_2_O (6 mL)^22^. The formation of the product was controlled by thin layer chromatography using MeOH/n-Hexane/EtOAc (1:1:1) as eluent. The obtained product was filtered, rinsed with deionized water several times, and dried at 100 °C. Finally, the recovery of the nanostructure from the reaction mixture was checked by using a centrifuge (2800 rpm for 7 min). After that, it was rinsed several times with deionized water and dried in an oven. Then, it was reused for reaction.

## Results and discussion

**Figure 2:**
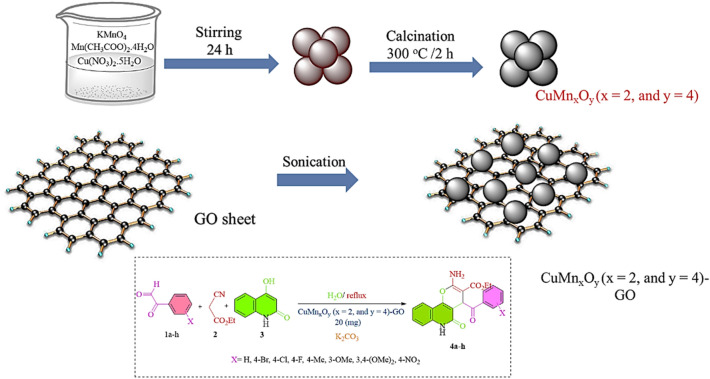
The stepwise synthesis route of CuMn_x_O_y_-GO nanocatalyst.

Based on our research in the development of nanostructures^26–31^. Many reports recorded on the preparation of pyranoquinolines using different catalysts^10,32–39^. In this study, the CuMn_x_O_y_-GO nanocatalyst was produced through a new strategy (Fig. 2). In this nanocatalyst, Cu and Mn nanoparticles have been grown on GO sheets. GO can be a good choice as a substrate due to its high electronic properties, easy preparation method, and large surface area for placing copper and manganese sites^40^. Then, CuMn_x_O_y_-GO was checked and confirmed through various methods. In the next step, this nanocatalyst was used in the synthesis and identification of pyranoquinolines. The distinguishing features of this work compared to previous works are cost-effectiveness, high efficiency, easy work-up, and recyclability of the nanocatalyst (Table 1).

**Table 1:** The catalytic efficacy comparison of CuMn_x_O_y_ (x = 2, and y = 4)-GO with other catalysts.

Entry	Catalyst	Condition	Time (h)	Yield (%)	References
1	Dioxane	Reflux	20	99	^41^
2	Cu-NP/C	80 °C	15	93	^32^
3	NH(Et)_2_	Solvent-free	10	83	^35^
4	TPAB	Reflux	5	91	^20^
5	Et_3_N	Reflux	4	82	^42^
6	DBU	Reflux	5 min	74	^43^
7	CuMn_x_O_y_ (x = 2, and y = 4)-GO	Reflux	2.5	96	Current study

### Characterization

The specifications of the devices used are explained in Text S2 of Support Information.

**Figure 3:**
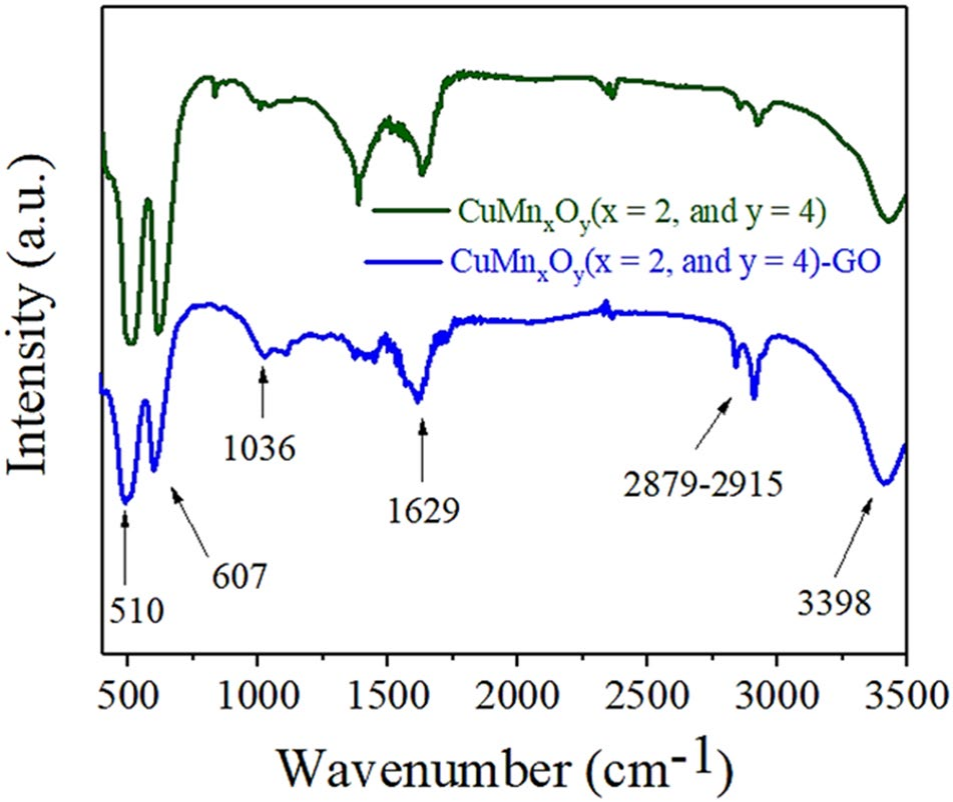
FT-IR spectrum of CuMn_x_O_y_ and CuMn_x_O_y_-GO nanocatalyst.

The FT-IR analysis was performed to investigate the intermolecular bonds and identify the type of functional groups present in the synthesized nanocatalyst (Fig. 3). The peaks around 510–607 cm^−1^ can be allocated to Cu–O and Mn–O bonds^44^. The CH_2_ stretching peaks were detected at 2879–2915 cm^−1^. The peaks were observed at 1036 and 1629 cm^−1^ corresponding to C–O–C and C=C, respectively. Also, the broad peak at 3398 cm^−1^ indicates the hydroxyl group, which is due to the absorption of a large amount of water on the nanocatalyst surface^45^.

**Figure 4:**
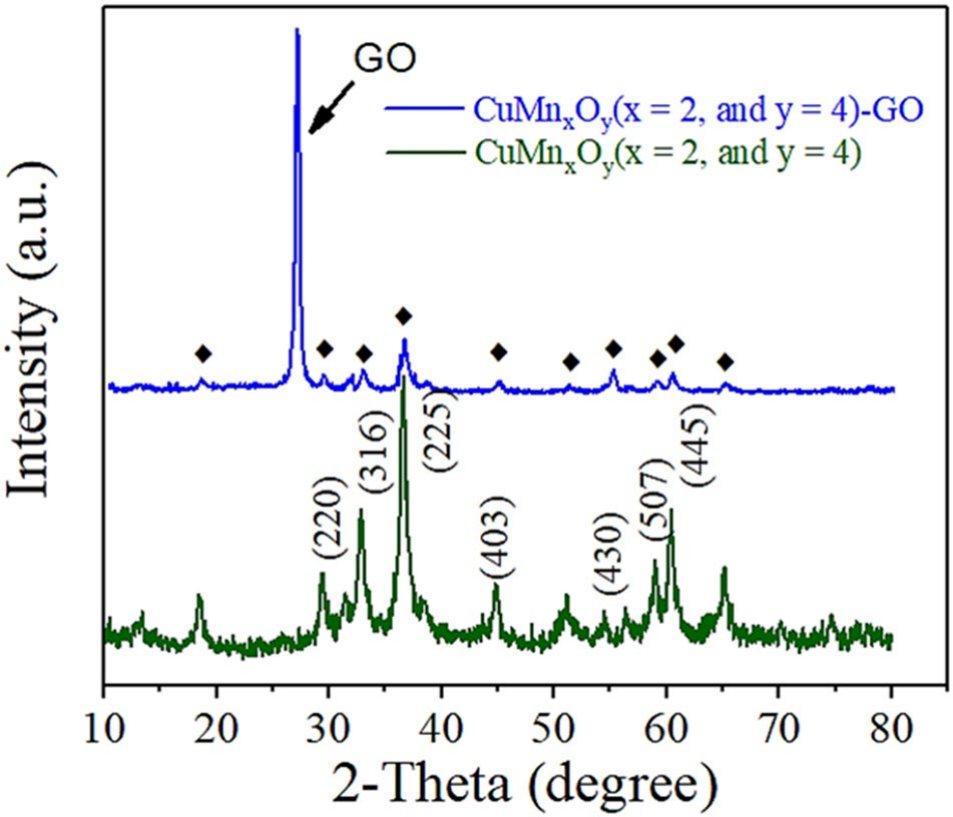
XRD spectrum of CuMn_x_O_y_ and Cu_x_MnO_y_-GO nanocatalyst.

The XRD pattern was used to identify the crystallinity and mineral phase of the CuMn_x_O_y_ and CuMn_x_O_y_-GO nanocatalyst (Fig. 4). The pattern involves the five considerable various peaks at 30.8°, 36.1°, 55.2°, 58.2°, and 63.04° could be ascribed to (220), (316), (430), (507), and (445) in CuMn_x_O_y_ crystal planes (JCPDS No. 84-0543)^46^, respectively. The peak at 26.72° can be assigned to GO in CuMn_x_O_y_-GO nanocatalyst^47^.

**Figure 5:**
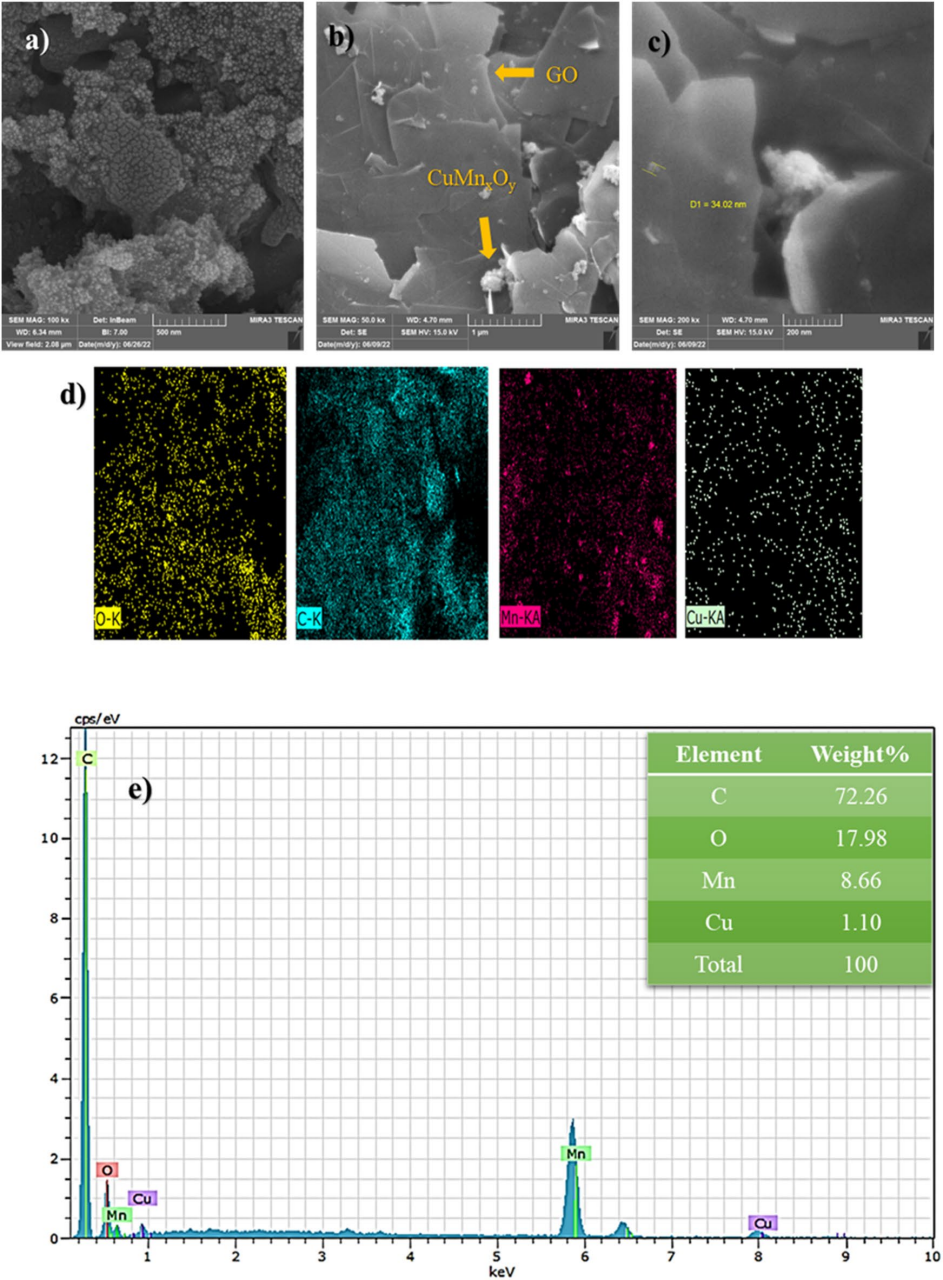
FESEM images of CuMn_x_O_y_ (**a**), and FESEM (**b** and **c**), SEM-mapping (**d**) and EDX (**e**) of CuMn_x_O_y_-GO.

Studies related to the morphology of the CuMn_x_O_y_ and CuMn_x_O_y_-GO nanocatalyst surfaces were conducted through FESEM analysis. The obtained results are shown in Fig. 5a–c. This analysis indicated images of the surface of CuMn_x_O_y_ nanoparticles and how they are placed between GO sheets. Also, SEM-mapping (Fig. 5d) and EDX (Fig. 5e) analyses were utilized to characterize the kind of elements and their percentage of abundance, respectively. Therefore, it was found that there are elements such as C, O, Mn, and Cu with different percentages in the composition of the nanostructure.

**Figure 6:**
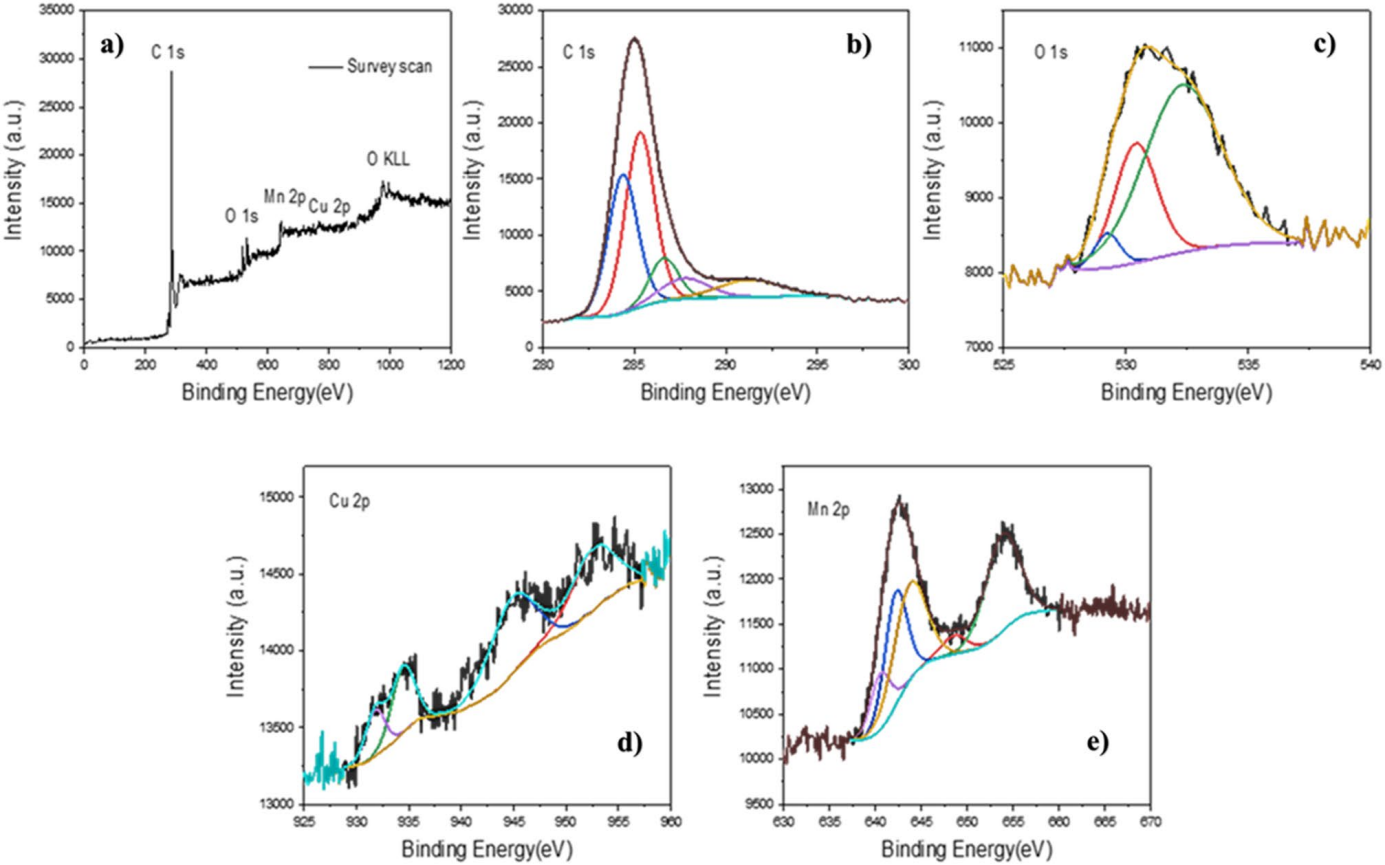
XPS spectra of the CuMn_x_O_y_-GO heterostructure: Survey scan (**a**), C 1s (**b**), O 1s (**c**), Cu 2p (**d**), and Mn 2p (**e**).

XPS analysis is one of the important and effective analyses to determine the type of bonds and the type of chemical composition in the structure of nanocatalysts^48^. According to the full scan XPS spectrum (survey) of the CuMn_x_O_y_-GO, the attendance of Cu, Mn, O, and C, atoms in the nanostructure is displayed in Fig. 6a, in the bond energies of 945.98 eV (Cu 2p), 643.38 eV (Mn 2p), 530.85 eV (O 1s), and 285.15 eV (C 1s) respectively^49^. The high-resolution spectrum of C 1s is divided into five peaks (Fig. 6b), which include energy bands of 291.40 eV are linked to the OH−C=O bond, 289.8 eV to the C=O bond, 286.78 eV to the C–O bond, 285.89 eV to the C=C bond, and 284.9 eV to the C−C bond of the GO compound^50^. However, the oxygen spectrum is converted to three peaks (Fig. 6c). The peak at 531.24 eV is attributed to the oxygens C–O/C=O/O–C=O in GO (O3), 530.89 eV is to the absorbed oxygens on the plane of CuMn_x_O_y_ (O2), and 529.46 eV is related to the lattice oxygens (O1)^51^.

The high-resolution XPS spectrum of Cu 2p is indicated in Fig. 6d. Two peaks at 957 eV and 936 eV matched Cu 2p1/2, and Cu 2p3/2. Cu in CuMn_x_O_y_ has oxidation states Cu^2+^ and Cu^1+^. The peak at 932.15 eV is attributed to Cu^1+^, while two peaks at 936.4 and 954.17 eV can correspond to Cu^2+^. In addition, the existence of a potent satellite peak at 946.28 eV indicates a high percentage of Cu^2+^ species^52^. The results obtained of the high-resolution spectrum of Mn 2p implied that the major peaks at 656 eV to Mn 2p_1/2_ and 646 eV are related to Mn 2p_3/2_ (Fig. 6e). The spectrum of Mn 2p_3/2_ fractures into three detached peaks at energies of 644.28, 642.7 and 641.16 eV, which respectively belong to Mn^4+^, Mn^3+^, and Mn^2+^ species in CuMn_x_O_y_-GO. However, the peak at 656.20 eV energy pertains to Mn^3+^ species sans any cleaving. Cu^1+^ is formed resulting from the equivalence reaction of ${\text{Mn}^{3+}}+ {\text{Cu}^{2+}}\longrightarrow {\text{Mn}^{4+}}+ {\text{Cu}^{+}}$. The atomic ratio of Mn/Cu is equal to 3.1, which discloses some surplus Mn on the surface of the nanocatalyst^53^.

**Figure 7:**
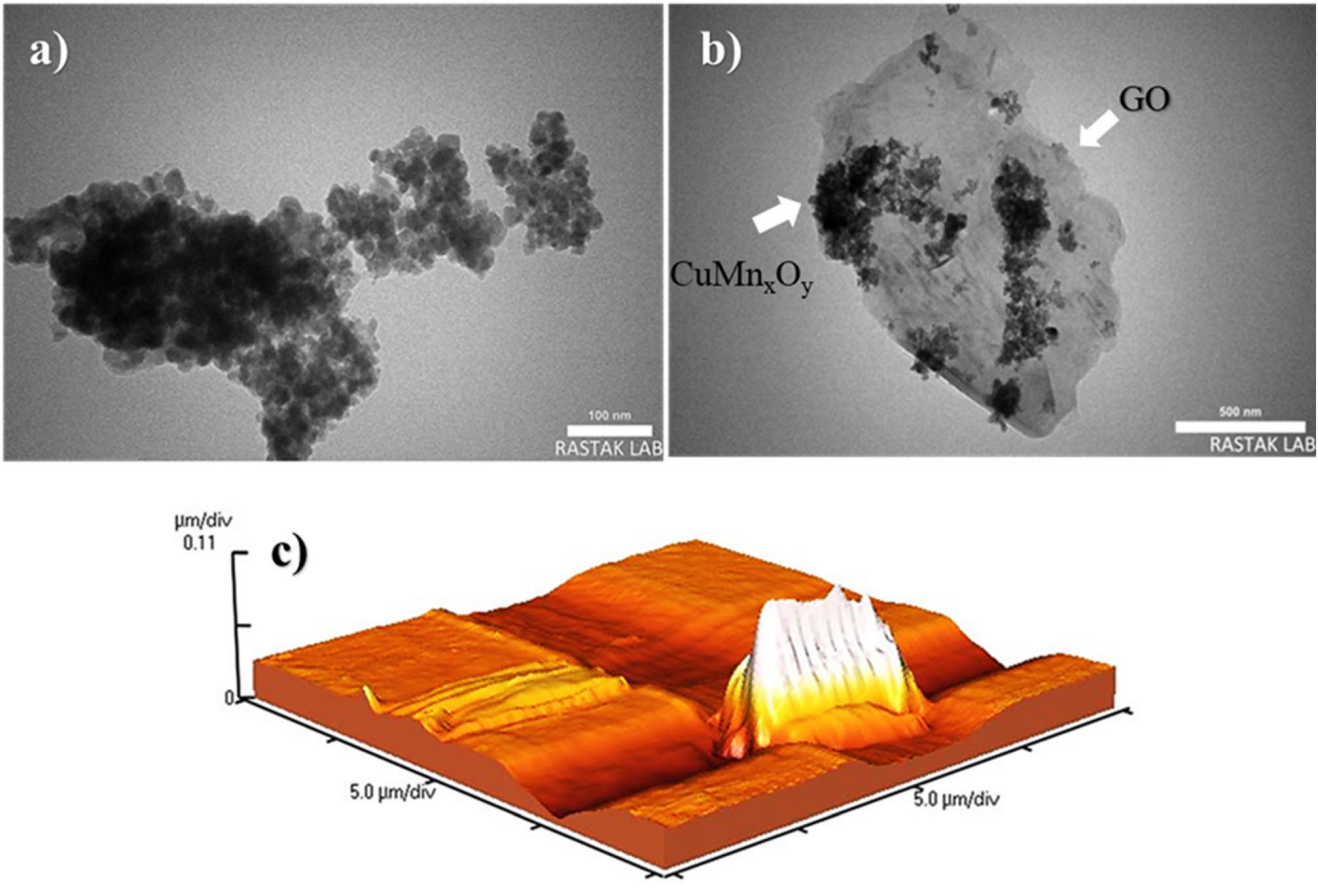
TEM images of CuMn_x_O_y_ (**a**), CuMn_x_O_y_-GO (**b**), and AFM of CuMn_x_O_y_-GO (**c**).

TEM spectrum was used to image the surface of the CuMn_x_O_y_ and CuMn_x_O_y_-GO with higher magnification (Fig. 7a,b). Also, this analysis has investigated the internal structure of the nanocatalyst. The images obtained from TEM analysis provided more and better details of the nanocatalyst surface, based on which the GO sheets are in the form of layers with spherical nanoparticles placed between them. AFM analysis was utilized to measure the dimensions of CuMn_x_O_y_-GO nanostructured plates. This analysis provided more information than FESEM about the morphology and roughness of the nanocatalyst surface (Fig. 7c). The obtained results showed that the nanoparticles are hill-like with a roughness size of 2.62 nm, which indicates the successful loading of nanoparticles on the GO plates.

### Optimization process

**Figure 8:**
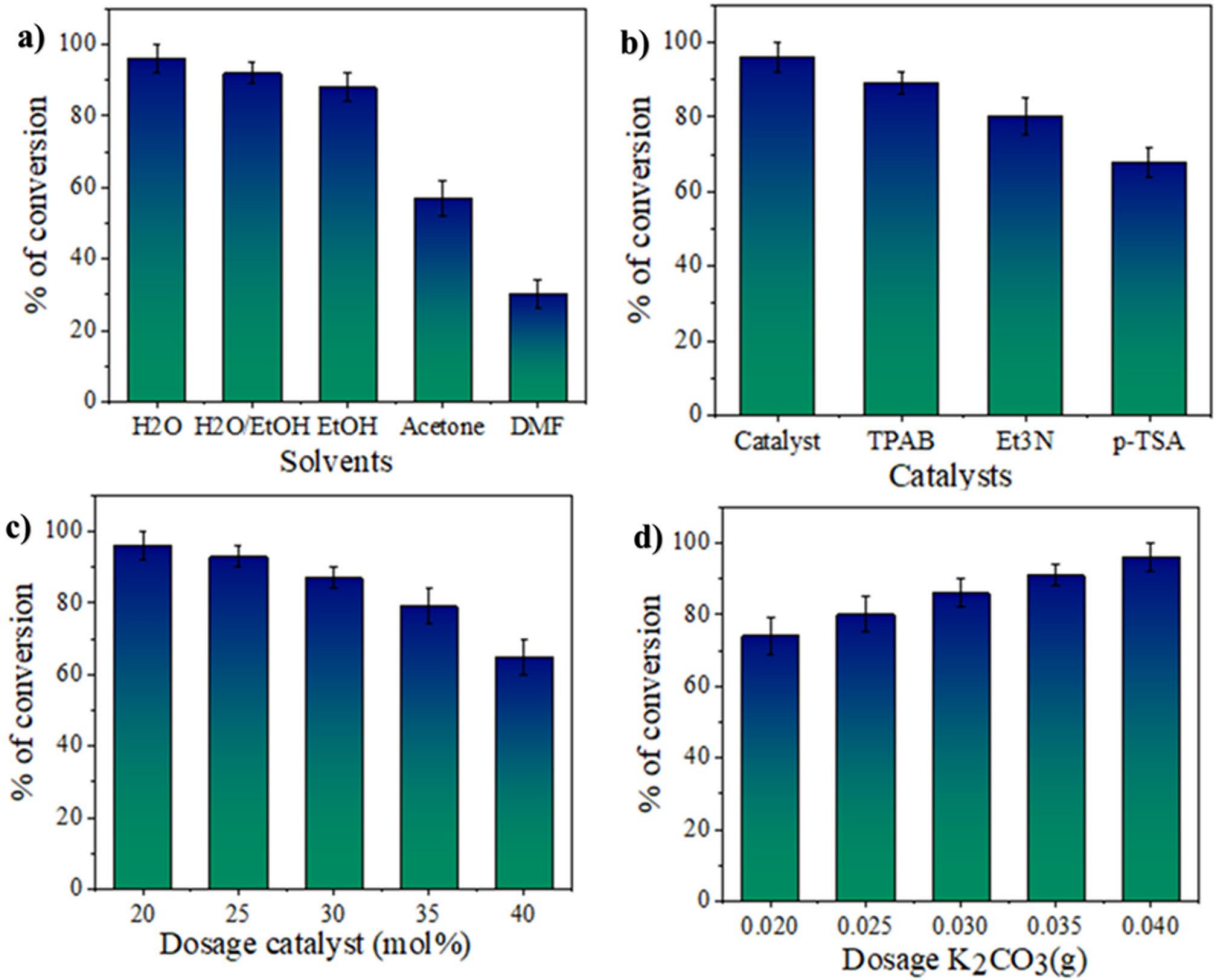
Optimization experiments for preparation of pyranoquinolines with CuMn_x_O_y_-GO nanocatalyst.

The synthesis process of pyranoquinolines was investigated using different solvents and catalysts under different conditions. The obtained results are shown below (Fig. 8). The effect of the CuMn_x_O_y_ was investigated in the reaction. The results displayed that the reaction proceeded at 4 h with 78% efficiency, while this reaction took place in the presence of CuMn_2_O_4_/GO nanocatalyst with 96% efficiency for 2.5 h. Meanwhile, the reaction did not proceed in the absence of the CuMn_x_O_y_-GO nanostructure. Also, the reaction was studied in the presence and absence of K_2_CO_3_ co-catalyst. The study indicated that the derivatives of pyranoquinolines were synthesized in the presence of K_2_CO_3_ and the absence of CuMn_x_O_y_-GO nanocatalyst with yield of 74% in 6 h. While this reaction took place in the presence of CuMn_x_O_y_-GO nanocatalyst and the absence of K_2_CO_3_ with an efficiency of 89% in 3.5 h. The effect of various solvents evaluated such as H_2_O, EtOH, H_2_O/EtOH, Acetone, and DMF (Fig. 8a). Derivatives of pyranoquinolines have been prepared in the presence of H_2_O with high efficiency compared to other solvents. The synthesis of pyranoquinolines using other catalysts was checked. As shown in Fig. 8b, the conversion of pyranoquinolines increased from 68 to 96% within 2.5 h, due to the presence of Cu and Mn active nanoparticles in GO sheets. Also, the impact dosage of nanocatalyst was checked from 20 to 40 mg on the prepared pyranoquinolines (Fig. 8c). In addition, the effective dosage of K_2_CO_3_ has been studied within the range of 20–40 mg (Fig. 8d). The most optimal conditions include the reaction of phenyl glyoxal (0.1 mmol, 10 mg), ethyl cyanoacetate (0.1 mmol, 10 mg), and 4-hydroxyquinoline (0.1 mmol, 16 mg) in the presence of CuMn_x_O_y_-GO nanocatalyst (20 mg), water (6 mL), and K_2_CO_3_ (0.3 mmol, 40 mg) in 2.5 h with 96% efficiency (Table 2).

**Table 2:**
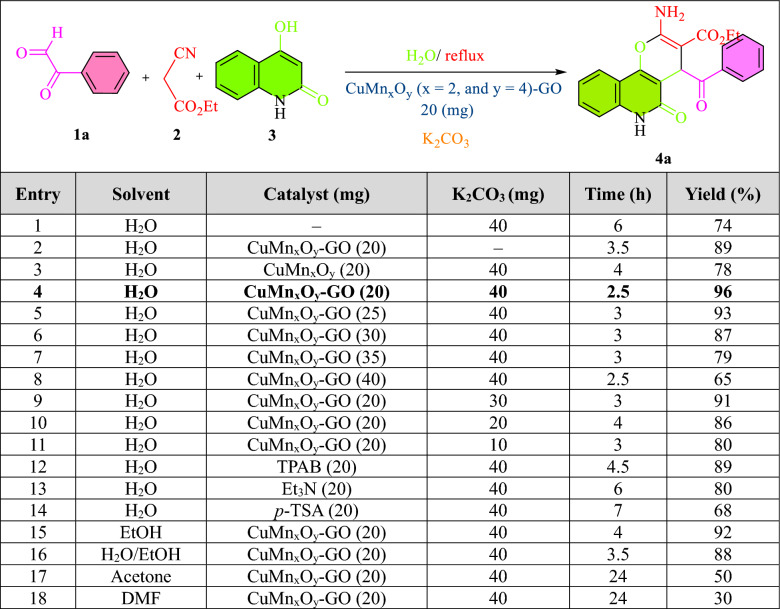
Impact of different parameters (Solvent, catalyst, and K_2_CO_3_) on the synthesis of compound **4a**.

Significant values are in [bold].

In the preparation of pyranoquinolines, the aryl glyoxals with different electron-donating and electron-withdrawing substituents were used. The structure of pyranoquinolines was investigated through different analyses (the results are shown in Text S3, Support Information). Among different derivatives, aryl glyoxal with H and NO_2_ substitution caused the synthesis of pyranoquinolines with 96% and 85% efficiency, respectively (Table 3). Meantime, the results displayed that the production of pyranoquinoline derivatives is not affected by the effects of electron-donating, electron-withdrawing, and steric hindrance of substitutions.

**Table 3:**
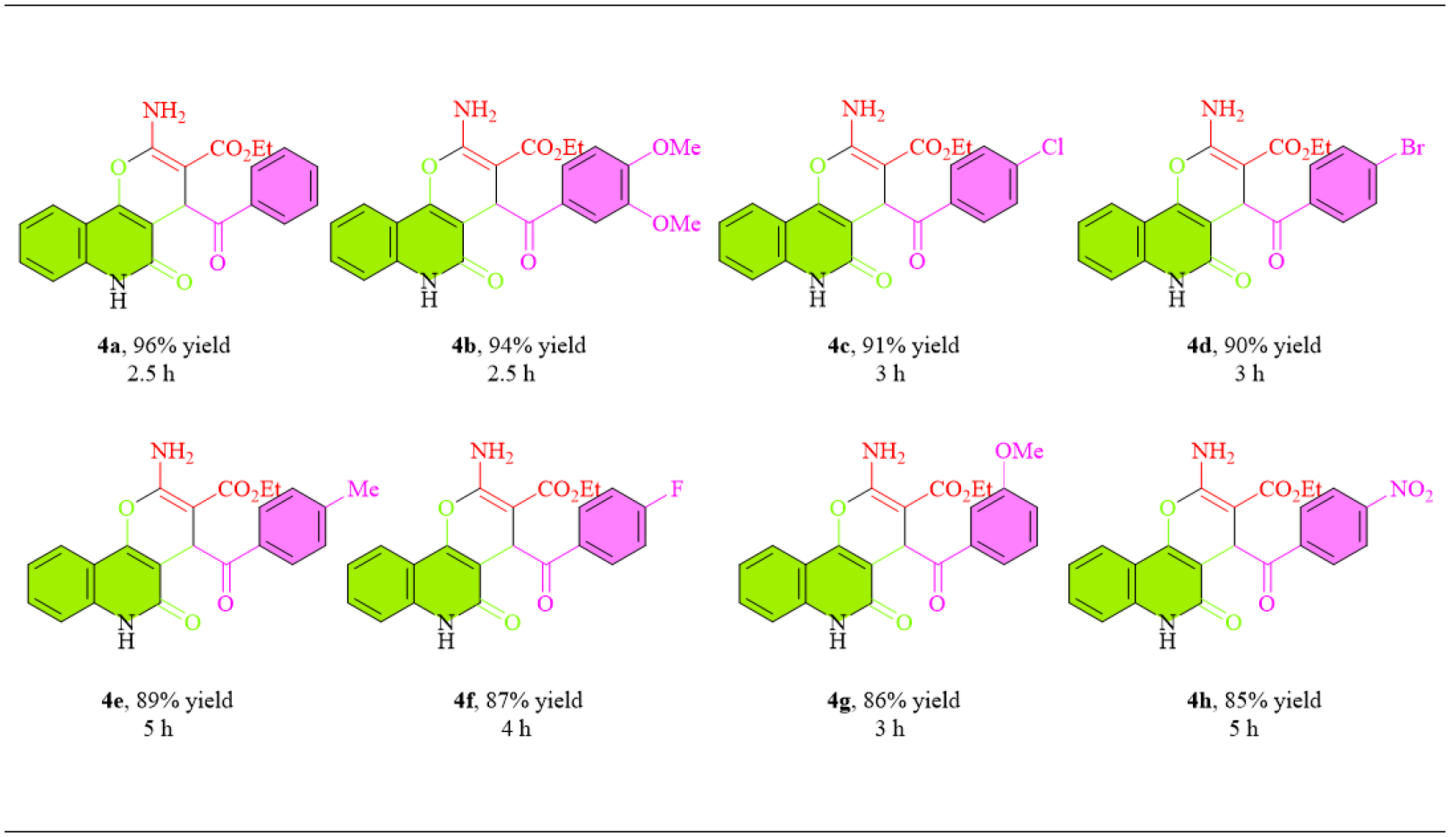
Prepare pyranoquinolines by CuMn_x_O_y_-GO under reflux conditions.

### Proposed mechanism of pyranoquinolines synthesis

**Figure 9:**
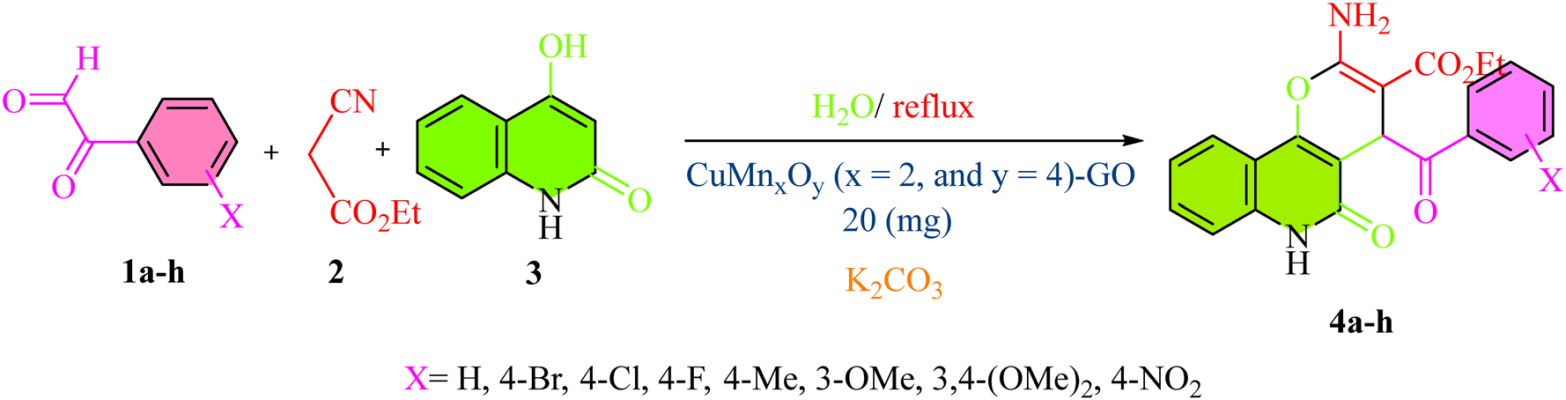
One-pot preparation of pyranoquinolines.

**Figure 10:**
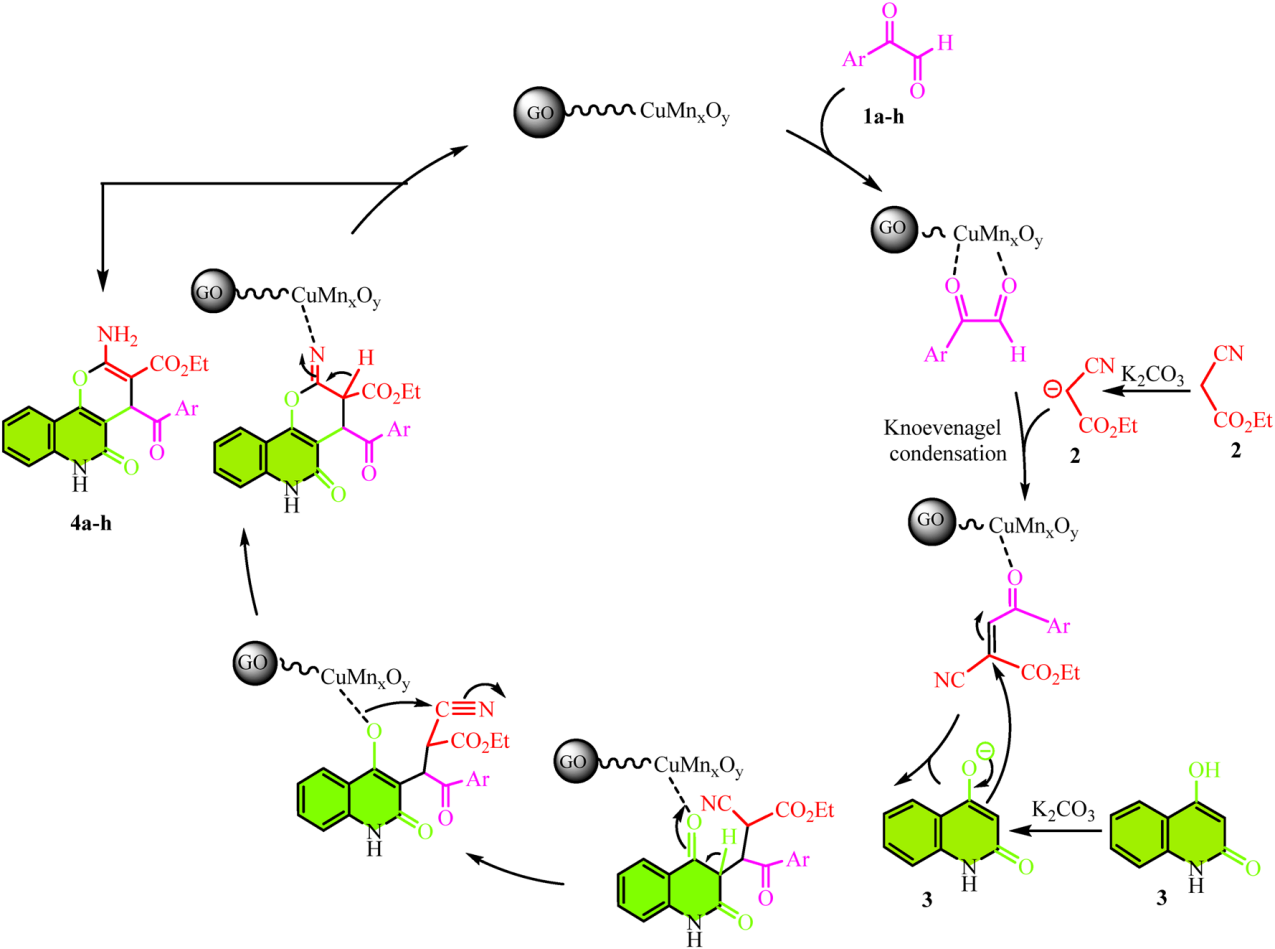
A plausible mechanism for the synthesis of pyranoquinolines **4a–h** using the CuMn_x_O_y_-GO.

The one-pot, three-component process between various aryl glyoxal derivatives, 4-hydroxyquinolin-2(1*H*)-one, ethyl cyanoacetate and using H_2_O, CuMn_x_O_y_-GO, and K_2_CO_3_ under reflux condition for synthesis pyranoquinolines with 85–96% yields as shown in Fig. 9. The proposed mechanism includes the coupling of copper and manganese active sites on the nanocatalyst with carbonyl groups in aryl glyoxal. Next, the acidic hydrogen of ethyl cyanoacetate is taken up by K_2_CO_3_. Then, *Knoevenagel* condensation was performed between aryl glyoxal and ethyl cyanoacetate as active methylene^54^. Afterwards, the hydrogen of the hydroxy group of 4-hydroxyquinoline is taken by K_2_CO_3_ and it reacted with the intermediate resulting from the *Knoevenagel* condensation. Finally, the process of cyclization and tautomerization leads to the preparation of desired derivatives **4a–h** in Fig. 10.

### Reusability studies

**Figure 11:**
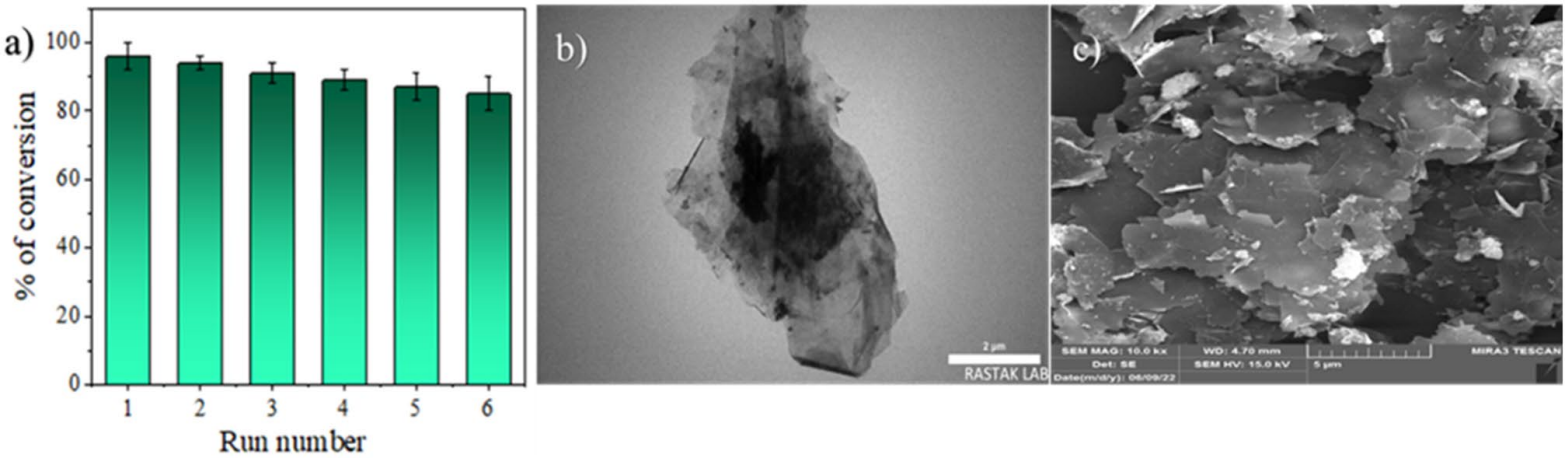
Reusability of CuMn_x_O_y_-GO nanocatalyst for synthesis of pyranoquinolines (**a**), TEM (**b**), and FESEM images of the CuMn_x_O_y_-GO after 6th recycle reaction (**c**).

Reusability ability is one of the important and effective factors in choosing the suitable catalyst for a chemical reaction. Here, the recycling process of the catalyst after the completion of the reaction was investigated by separating it from the reaction mixture using a centrifuge (2800 rpm for 7 min) then washing it with deionized water, and finally drying it in an oven. The CuMn_x_O_y_-GO was recovered during 6 cycles. The results obtained are shown below (Fig. 11a). During the recycling process, no significant change in catalyst activity was observed. The images obtained through TEM (Fig. 11b), and FESEM (Fig. 11c) analyses showed that the structure of the catalyst is almost stable.

## Conclusions

In this study, the CuMn_x_O_y_-GO nanocatalyst has been prepared by loading CuMn_x_O_y_ nanoparticles between GO sheets. The obtained results from the CuMn_x_O_y_-GO of the structure indicated that its synthesis was successful. Pyranoquinoline derivatives have been synthesized using CuMn_x_O_y_-GO nanocatalyst. The high activity of the catalyst causes a fast and effective reaction between aryl glyoxal, 4-hydroxyquinolin-2(1*H*)-one, and ethyl cyanoacetate in the presence of K_2_CO_3_, water and produced the desired derivatives with high yields. The features of this work include the use of water as a green solvent, economics, the reusability of nanocatalyst after several times without losing its catalytic activity, and high yields of products. In the future, this work can be a good model for the synthesis of heterocyclic derivatives in the presence of nanocatalysts based on carbon material.

## Supplementary Information

Supplementary Information.

## Data Availability

All data generated or analyzed during this study are included in this published article [and its supplementary information files].
