# Supplementary material for: Development of CuMnxOy (x = 2, and y = 4)-GO heterostructure for the synthesis of pyranoquinoline derivatives
Source: Sci Rep. 2023 Jun 21;13:10112. doi: 10.1038/s41598-023-36529-y (PMC10284822; doi:10.1038/s41598-023-36529-y)
Supplement: Supplementary file 1 — Supplementary Information. [file 41598_2023_36529_MOESM1_ESM.pdf]

Supporting Information

Development of $CuMn_xO_y$ (x = 2, and y = 4)-GO heterostructure for the synthesis of pyranoquinoline derivatives

Ayda Farajollahi, Nader Noroozi Pesyan*, Ahmad Poursattar Marjani*, and Hassan Alamgholiloo

Department of Organic Chemistry, Faculty of Chemistry, Urmia University, Urmia, Iran

*E-mail: n.noroozi@urmia.ac.ir ; a.poursattar@urmia.ac.ir

Table of content

Text S1. Prepare GO nanosheets

Text S2. Characterization

Text S3. Characterization of as-produced derivatives

Fig. S1. ^{1}H NMR spectrum of compound **4a**.

Fig. S2. ^{13}C NMR spectrum of compound **4a**

Fig. S3. IR spectrum of compound **4a**

Fig. S4. ^{1}H NMR spectrum of compound **4b**

Fig. S5. ^{13}C NMR spectrum of compound **4b**

Fig. S6. IR spectrum of compound **4b**

Fig. S7. ^{1}H NMR spectrum of compound **4c**

Fig. S8. ^{13}C NMR spectrum of compound **4c**

Fig. S9. IR spectrum of compound **4c**

Fig. S10. ^{1}H NMR spectrum of compound **4d**

Fig. S11. ^{13}C NMR spectrum of compound **4d**

Fig. S12. IR spectrum of compound **4d**

Fig. S13. ^{1}H NMR spectrum of compound **4e**

Fig. S14. ^{13}C NMR spectrum of compound **4e**

Fig. S15. IR spectrum of compound **4e**

Fig. S16. ^{1}H NMR spectrum of compound **4f**

Fig. S17. ^{13}C NMR spectrum of compound **4f**

Fig. S18. IR spectrum of compound **4f**

Fig. S19. ^{1}H NMR spectrum of compound **4g**

Fig. S20. ^{13}C NMR spectrum of compound **4g**

Fig. S21. IR spectrum of compound **4g**

Fig. S22. ^{1}H NMR spectrum of compound **4h**

Fig. S23. ^{13}C NMR spectrum of compound **4h**

Fig. S24. IR spectrum of compound **4h**

References

Text S1. Prepare GO nanosheets

The GO was fabricated from graphite through the Hummers method [S1-S3]. In this method, graphite was oxidized by using potassium permanganate and sodium nitrate in concentrated sulfuric acid to form graphite oxide. After that, prepared graphite oxide was delaminated into GO nanosheets by surfactant of poly(ethylene glycol)-block-poly(propylene glycol)-block-poly (ethylene glycol) P123 surfactant/ethylene glycol (EG) (1:10) under ultrasonic irradiation. The synthesized black precipitate was diluted 10 times and the electrical conductivity fabricated (5.85 ± 0.3) × 10^3 S m^{-1} at ambient conditions, which compared to that of formerly reported data for GO.

Text S2. Characterization

Fourier transform infrared (FT-IR) spectra were obtained using a Shimadzu IR-640 spectrometer, and absorbencies are reported in cm^{-1}. ^1H and ^{13}C nuclear magnetic resonance (NMR) spectra were recorded with a Burker spectrometer at 300 and 75 MHz, respectively (Burker, Billerica, MA). The melting points of all the prepared derivatives were measured with an Electrothermal 9100 device. The crystalline phases of the $CuMn_xO_y$ (x = 2, and y = 4)-GO nanocatalyst were identified by X-ray powder diffraction (XRD) measurements (Philips-PW 1730 diffractometer). Field Emission Scanning Electron Microscope (FE-SEM) and SEM-mapping images of samples prepared by using a TESCAN MIRA3 microscope. The type of bonds and chemical composition has been determined by X-ray Photoelectron Spectroscopy (XPS) (SPECS, UHV analysis system). Transmission electron microscopy (TEM) images were recorded with a Philips EM 208S electron microscope. The images of the surface roughness of the nanostructure were obtained using the Atomic Force Microscope (AFM) (Dual Scope TMDS 95-200l50).

Text S3. Characterization of as-produced derivatives

Ethyl 2-amino-4-benzoyl-5-oxo-5,6-dihydro-4*H*-pyrano[3,2-*c*]quinoline-3-carboxylate (4a). Yield, 96%; white powder; mp 223-226 ºC (S4). ν_{max} (KBr): 3405, 3300, 2975, 2849, 1676, 1529, 1375, 1272, 1220, 1084, 747 cm^{-1}; δ_H (300 MHz, DMSO-*d₆*) 11.75 (s, 1H, NH, exchanged by D_2O addition), 8.16 (d, 2H, *J* = 6.6 Hz, ArH), 7.96 (t, 1H, *J* = 7.8 Hz, ArH), 7.93 (s, 2H, NH_2, exchanged by D_2O addition), 7.57-7.66 (m, 3H, ArH), 7.54 (d, 2H, *J* = 7.8 Hz, ArH), 7.31-7.36 (m,1H, ArH),

5.67 (s, CH, 1H), 3.77 (bq, 2H, CH_2), 0.68 (bt, 3H, CH_3); δc (75 MHz, DMSO-d_6) 194.1, 167.9, 161.8, 160.3, 153.1, 138.2, 136.7, 132.8, 130.7, 130.2, 129.5, 128.4, 123.5, 117.2, 115.0, 114.8, 112.7, 110.1, 73.8, 14.9.

Ethyl 2-amino-4-(3,4-dimethoxybenzoyl)-5-oxo-5,6-dihydro-4*H*-pyrano[3,2-*c*]quinoline-3-carboxylate (4b).

Yield, 94%; creme powder; mp 230-233 ºC (S4). ν_{max} (KBr): 3390, 3300, 2950, 2845, 1673, 1518, 1381, 1254, 1164, 1094, 1028, 766 cm^{-1}; δ_H (300 MHz, DMSO-d_6) 11.71 (s, 1H, NH, exchanged by D_2O addition), 7.91-7.84 (m, 2H, ArH) 7.88 (s, 2H, NH_2, exchanged by D_2O addition), 7.65 (s, 1H, ArH), 7.56-7.58 (m, 1H, ArH), 7.30-7.36 (m, 2H, ArH), 7.11-7.13 (m, 2H, ArH), 5.63 (s, 1H, CH), 3.86 (s, 3H, OCH_3), 3.85 (q, J = 8.1 Hz, 2H, CH_2), 3.80 (s, 3H, OCH_3), 0.92 (t, J = 8.1 Hz, 3H, CH_3); δc (75 MHz, DMSO-d_6) 193.6, 168.3, 162.2, 160.6, 153.2, 148.8, 139.3, 130.3, 126.3, 123.4, 123.2, 115.8, 114.8, 113.6, 112.4, 110.9, 110.0, 109.9, 74.5, 56.3, 55.3, 14.9.

Ethyl 2-amino-4-(4-chlorobenzoyl)-5-oxo-5,6-dihydro-4*H*-pyrano[3,2-*c*]quinoline-3-carboxylate (4c).

Yield, 91%; white powder; mp 242-245 º C (S4). ν_{max} (KBr): 3404, 3294, 2957, 2839, 1672, 1593, 1526, 1380, 1271, 1089, 757 cm^{-1}; δ_H (300 MHz, DMSO-d_6) 11.73 (s, 1H, NH, exchanged by D_2O addition), 8.20 (d, J = 7.8 Hz, 2H, ArH), 7.93-7.95 (m, 1H, ArH), 7.92 (2H, NH_2, exchanged by D_2O addition), 7.60 (d, J = 8.7 Hz, 2H, ArH), 7.51-7.57 (m, 1H, ArH), 7.35 (d, 1H, J = 9.3 Hz, ArH), 7.34 (t, 1H, J = 7.2 Hz, ArH), 5.56 (s, 1H, CH), 3.75 (q, 2H, J = 6.6 Hz, CH_2), 0.73 (t, 3H, J = 6.6 Hz, CH_3); δ_c (75 MHz, DMSO-d_6) 196.6, 168.2, 161.5, 160.7, 153.4, 138.2, 136.9, 132.4, 130.7, 127.5, 123.7, 122.3, 121.5,116.9, 115.0, 114.8, 112.6, 109.2, 73.5, 14.8.

Ethyl 2-amino-4-(4-bromobenzoyl)-5-oxo-5,6-dihydro-4*H*-pyrano[3,2-*c*]quinoline-3-carboxylate (4d).

Yield, 90%; yellow powder; mp 238-240 ºC (S4). ν_{max} (KBr): 3403, 3292, 2967, 2851, 1671, 1525, 1379, 1274, 1084, 755 cm^{-1}; δ_H (300 MHz, DMSO-d_6) 11.74 (s, 1H, NH, exchanged by D_2O addition), 7.96 (d, 2H, J = 8.4 Hz, ArH), 7.91-7.93 (m,1H,ArH), 7.94 (s,2H, NH_2, exchanged by D_2O addition), 7.75 (d, 2H, J = 8.4 Hz, ArH), 7.66 (d, 1H, J = 8.4 Hz, ArH), 7.61 (t, 1H, J = 8.1 Hz, ArH), 7.49(d, 1H, J= 8.1 Hz, ArH), 5.57 (s, 1H, CH), 3.78 (q, 2H, J = 6.6 Hz, CH_2), 0.72 (t,

3H, J = 6.9 Hz, CH_3); δc (75 MHz, DMSO-d_6) 197.7, 176.5, 168.3, 161.5, 160.7, 152.7, 142.6, 140.9, 138.2, 137.4, 132.7, 132.8, 130.2, 126.9, 123.6, 112.6, 109.6, 106.1 ,73.9, 14.7.

Ethyl 2-amino-4-(4-methylbenzoyl)-5-oxo-5,6-dihydro-4*H*-pyrano[3,2-*c*]quinoline-3-carboxylate (4e).

Yield, 89%; white powder; mp 239-242 ºC (S4). ν_{max} (KBr): 3387, 3288, 2945, 2847, 1672, 1536, 1377, 1273, 1179, 1087, 1024, 748 cm^{-1}; δ_H (300 MHz, DMSO-d_6) 11.72 (s, 1H, exchanged by D_2O addition, NH), 8.09 (d, J = 8.4 Hz, 2H, ArH), 7.95 (t, J = 6.9 Hz, 1H, ArH), 7.91 (s, 2H, NH_2, exchanged by D_2O addition), 7.54 (t, 1H, J = 8.1 Hz, ArH), 7.37(d, J = 8.1 Hz, 1H, ArH), 7.32 (d, J = 7.2 Hz, 2H, ArH), 7.29-7.33 (m, 1H, ArH), 5.62 (s, 1H, CH), 3.74 (q, J = 7.2 Hz, 2H, CH_2), 0.75 (t, 3H, J = 6.9 Hz, CH_3); δc (75 MHz, DMSO-d_6) 192.4, 168.1, 161.6, 160.3, 152.0, 144.5, 138.4, 138.1, 135.2, 130.9, 130.8, 128.7, 128.1, 125.5, 116.9, 114.9, 112.8, 109.9, 73.9, 21.55, 14.7.

Ethyl 2-amino-4-(4-fluorobenzoyl)-5-oxo-5,6-dihydro-4*H*-pyrano[3,2-*c*]quinoline-3-carboxylate (4f).

Yield, 87%; white powder; mp 243-246 ºC (S4). ν_{max} (KBr): 3409, 3304, 2976, 2853, 1674, 1513, 1382, 1275, 1221, 1089, 757 cm^{-1}; δ_H (300 MHz, DMSO-d_6) 11.65 (s, 1H, NH, exchanged by D_2O addition), 8.21 (d, 2H, J = 7.8 Hz, ArH), 7.94 (2H, NH_2, exchanged by D_2O addition), 7.73 (d, J = 8.7 Hz, 2H, ArH), 7.59 (1H, d, J = 7.2 Hz, ArH), 7.47 (d, 1H, J= 8.1 Hz, ArH), 7.32 (t, 1H, J = 7.2 Hz, ArH), 7.12-7.31 (m, 2H, ArH), 5.64 (s, 1H, CH), 3.75 (q, 2H, J = 7.2 Hz, CH_2), 0.74 (t, 3H, J = 8.7 Hz, CH_3); δc (75 MHz, DMSO-d_6) 194.8, 168.3, 164.1, 162.8, 161.8, 160.6, 152.7, 139.9, 138.4, 135.1, 131.4, 123.5, 122.3, 121.9, 116.1, 114.6, 112.7, 109.6, 73.8, 14.7.

Ethyl 2-amino-4-(3-methoxybenzoyl)-5-oxo-5,6-dihydro-4*H*-pyrano[3,2-*c*]quinoline-3-carboxylate (4g).

Yield, 86%; white powder; mp 227-229 ºC (S4). ν_{max} (KBr): 3407, 3303, 2965, 2837, 1685, 1592, 1525, 1381, 1262, 1087, 759 cm^{-1}; δ_H (300 MHz, DMSO-d_6) 11.69 (s, 1H, NH, exchanged by D_2O addition), 7.94 (t, 1H, J = 7.8 Hz, ArH), 7.92 (s, 2H, NH_2, exchanged by D_2O addition), 7.76 (d, 1H, Ar, J = 7.8 Hz), 7.61 (s, 1H, ArH), 7.59 (t, 1H, J = 7.6 Hz, ArH), 7.49 (t, 1H, J = 7.8 Hz, ArH), 7.25-7.39 (m, 2H, ArH), 7.20-7.25 (m, 1H, ArH), 5.59 (s, 1H, CH), 3.85 (s, 3H, OCH_3), 3.81 (q, 2H, CH_2, J = 6.9 Hz, CH_2), 0.75 (3H, CH_3, J = 6.9 Hz); δc (75 MHz, DMSO-d_6) 198.6, 168.3,

161.8, 160.6, 159.4, 153.2, 139.3, 139.2, 138.6, 133.1, 130.6, 128.5, 123.2, 121.5, 119.8, 114.9, 112.8, 109.8, 73.9, 55.3, 14.7.

Ethyl 2-amino-4-(4-nitro)-5-oxo-5,6-dihydro-4*H*-pyrano[3,2-*c*]quinoline-3-carboxylate (4h).

Yield, 85%; brown powder; mp 223-226 ºC (S4). ν_{max} (KBr): 3407, 3296, 2980, 2856, 1523, 1681, 1347, 1281, 1226, 1095, 756 cm^{-1}; δ_H (300 MHz, DMSO-d_6), 11.78 (s, 1H, NH, exchanged by D_2O addition), 8.40 (d, J = 7.5 Hz, 2H, ArH), 8.34-8.38 (m, 1H, ArH), 7.98 (s, 2H, NH_2, exchanged by D_2O addition), 7.90-7.95 (m, 1H, ArH), 7.57-7.62 (m, 2H, ArH), 7.29-7.38 (m, 2H, ArH), 5.66 (s, CH, 1H), 3.76 (q, 2H, J = 7.8 Hz, CH_2), 0.67 (t, 3H, J = 7.2 Hz, CH_3); δ_C (75 MHz, DMSO-d_6) 194.6, 168.1, 164.93, 162.8, 161.7, 160.6, 153.2, 139.7, 138.2, 134.7, 131.4,123.5, 122.0, 121.9, 116.3, 114.6, 112.6, 109.6, 73.8, 14.9.

Ethyl 2-amino-4-benzoyl-5-oxo-5,6-dihydro-4*H*-pyrano[3,2-*c*]quinoline-3- carboxylate (4a).

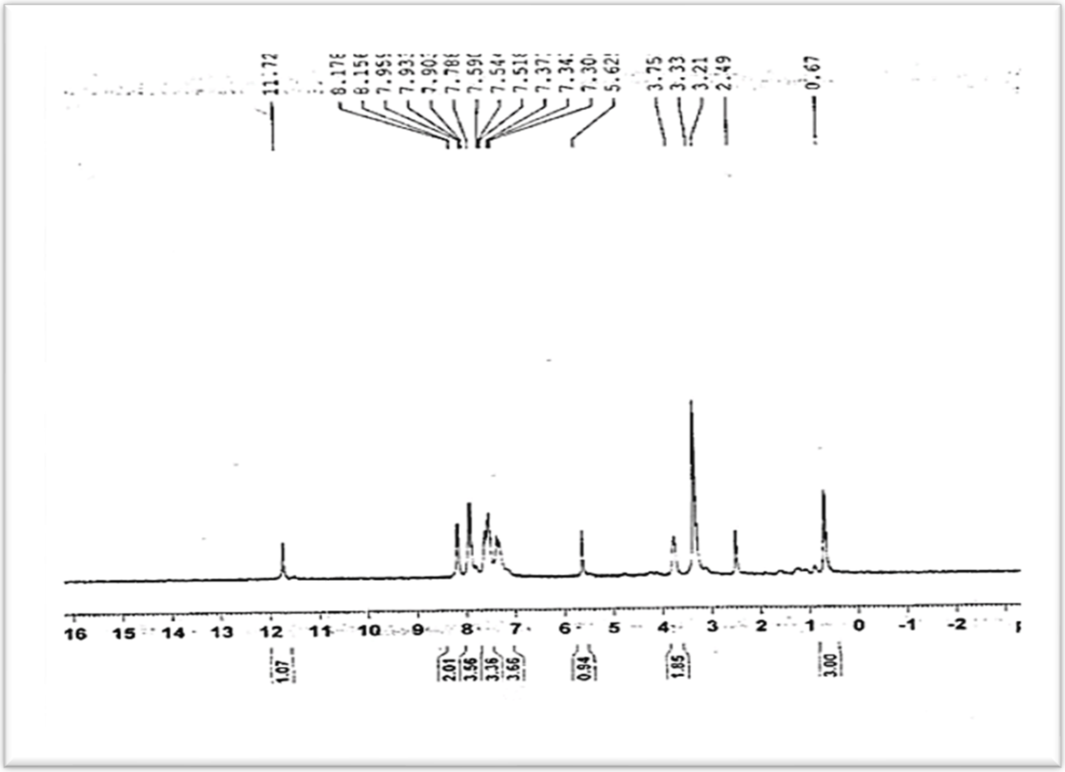

Fig. S1. ^{1}H NMR spectrum of compound **4a.**

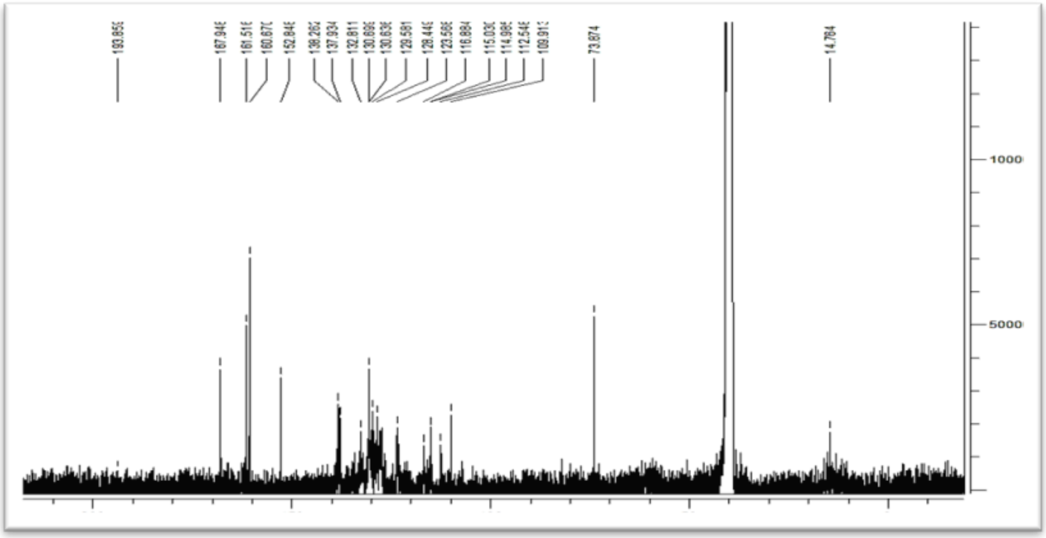

Fig. S2. ^{13}C NMR spectrum of compound **4a**.

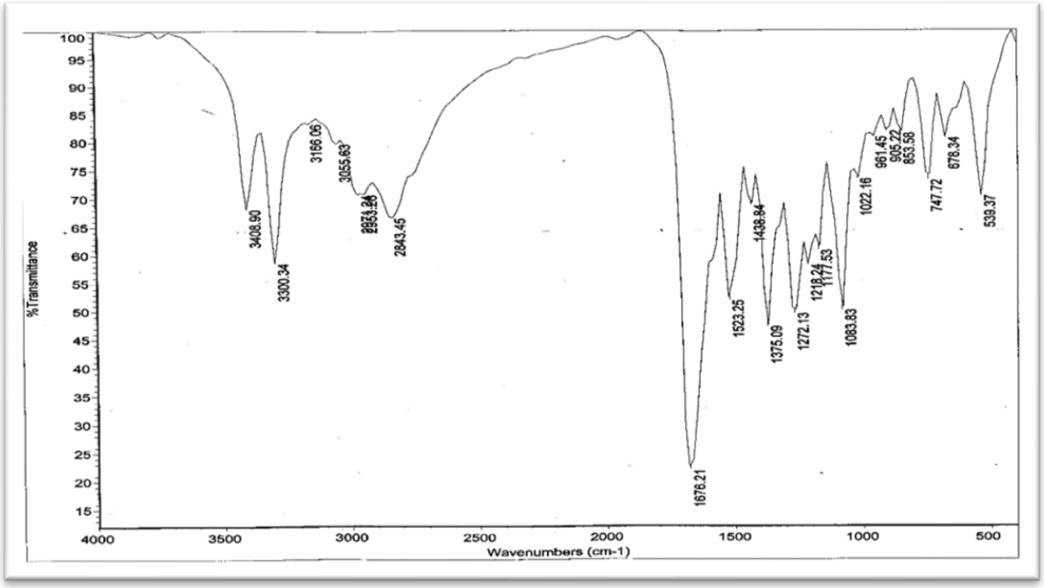

Fig. S3. IR spectrum of compound **4a**.

Ethyl 2-amino-4-(3,4-dimethoxybenzoyl)-5-oxo-5,6-dihydro-4*H*-pyrano[3,2-*c*]quinoline-3-carboxylate (4b)

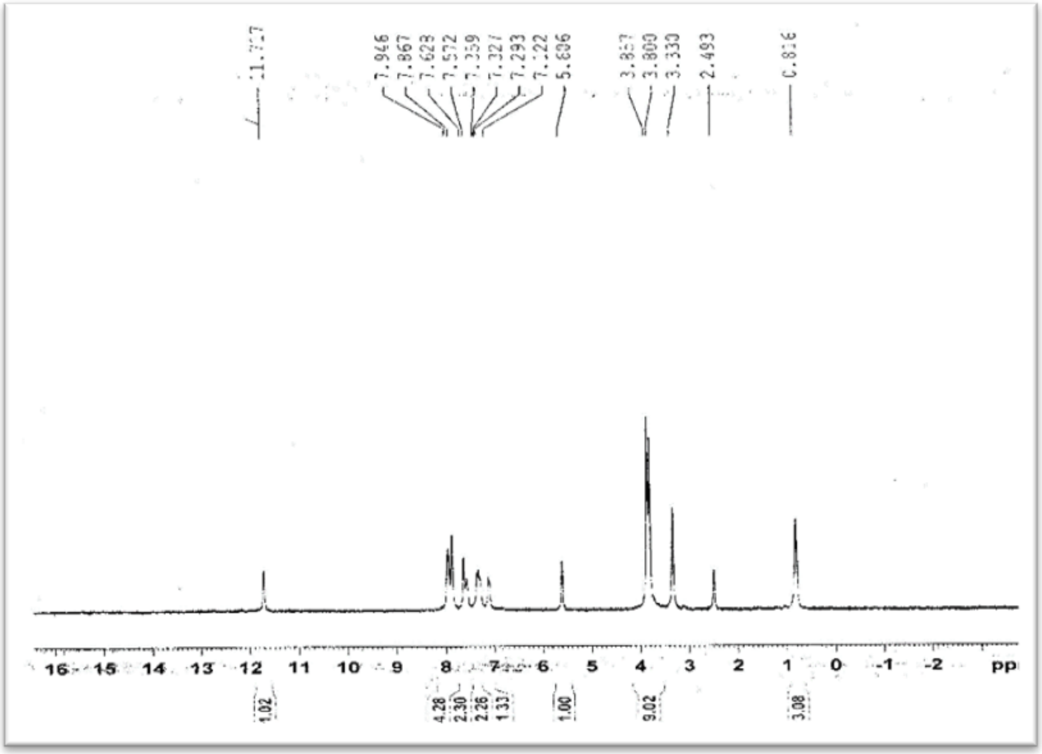

Fig. S4. ^{1}H NMR spectrum of compound **4b**.

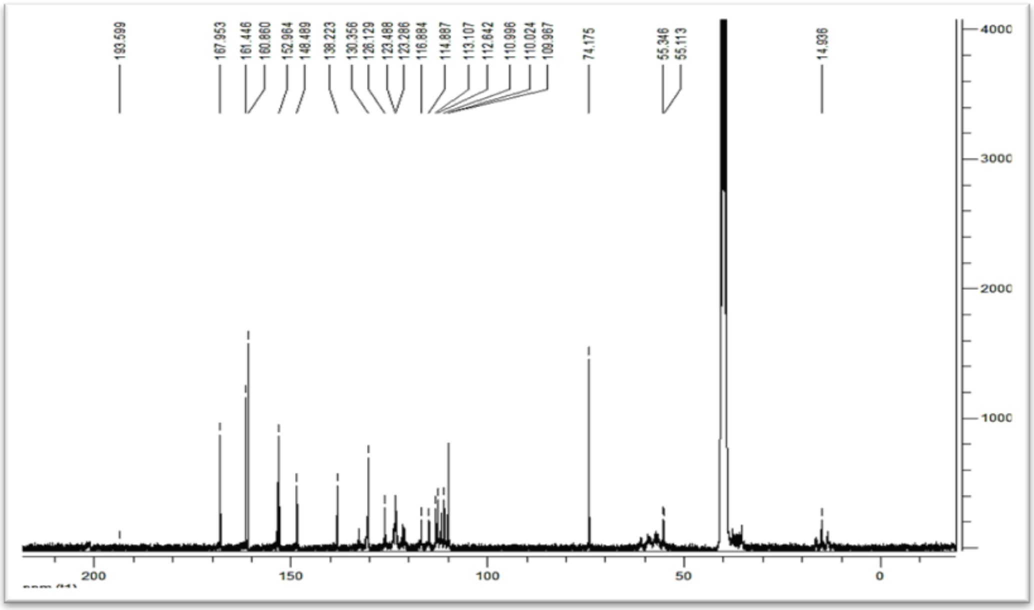

Fig. S5. ^{13}C NMR spectrum of compound **4b**.

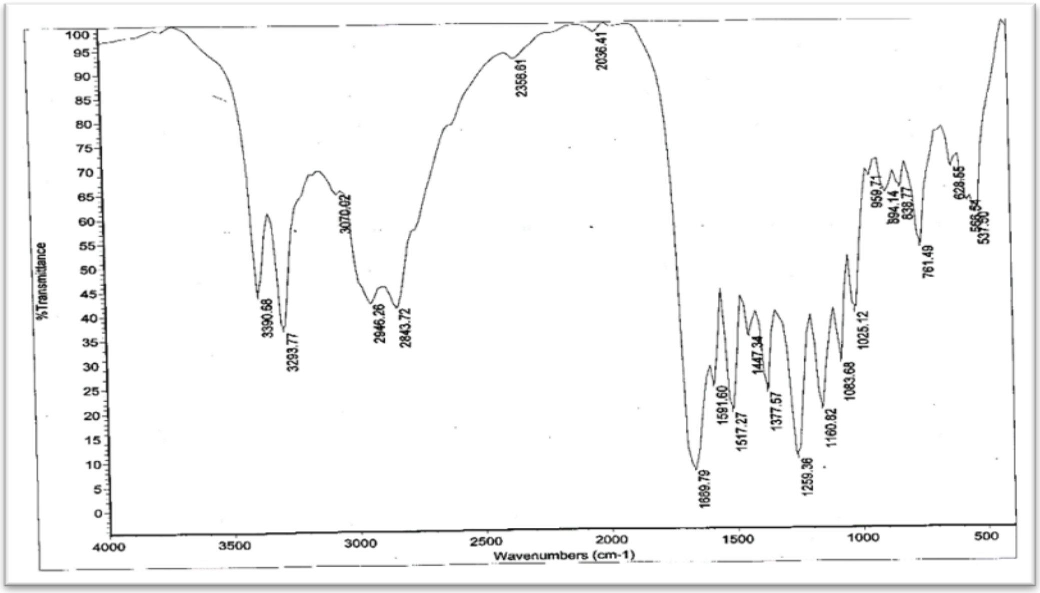

Fig. S6. IR spectrum of compound **4b**.

Ethyl 2-amino-4-(4-chlorobenzoyl)-5-oxo-5,6-dihydro-4*H*-pyrano[3,2-*c*]quinoline-3-carboxylate (4c)

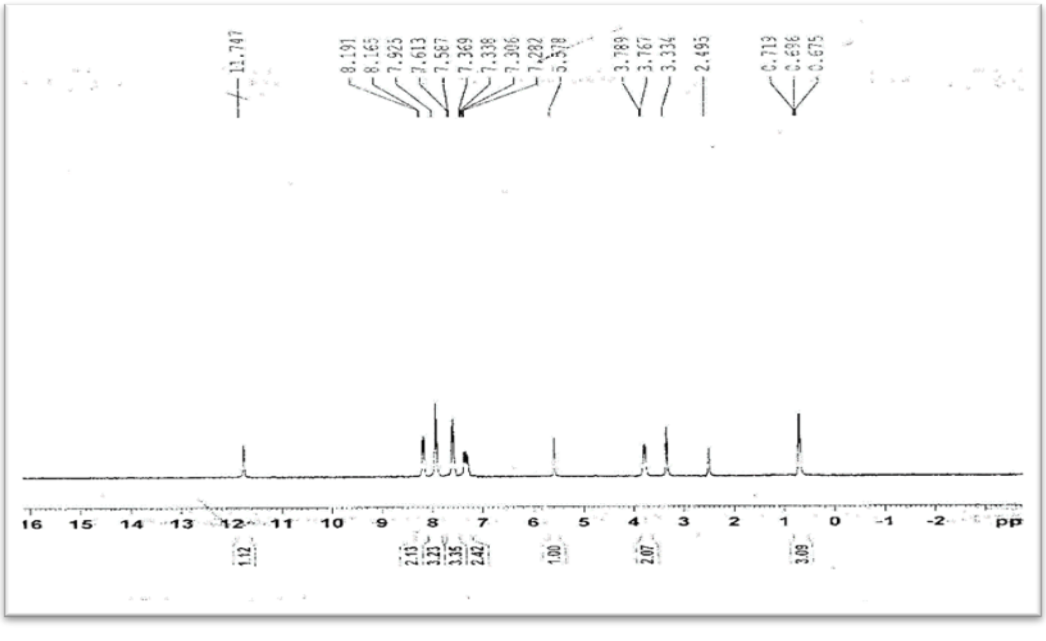

Fig. S7. ^{1}H NMR spectrum of compound **4c**.

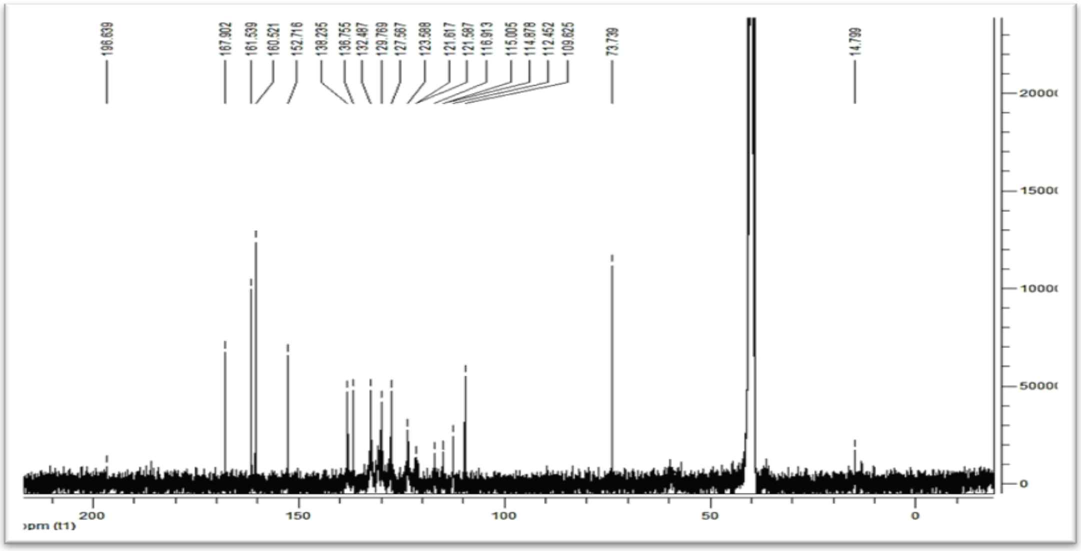

Fig. S8. ^{13}C NMR spectrum of compound **4c**.

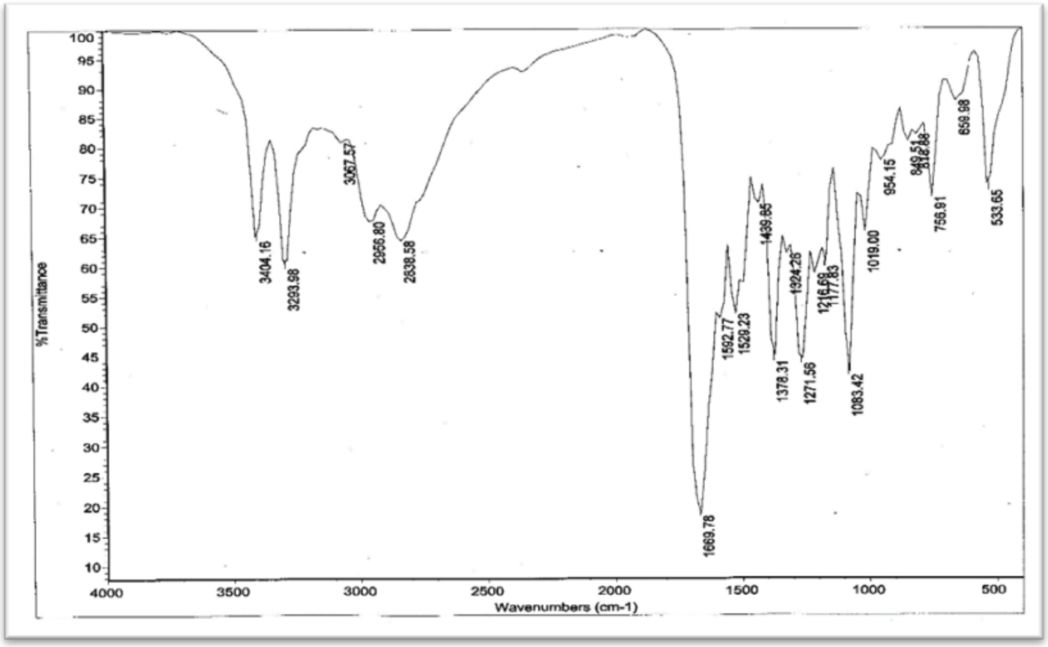

Fig. S9. IR spectrum of compound **4c**.

Ethyl 2-amino-4-(4-bromobenzoyl)-5-oxo-5,6-dihydro-4*H*-pyrano[3,2-c] quinoline-3-carboxylate (4e)

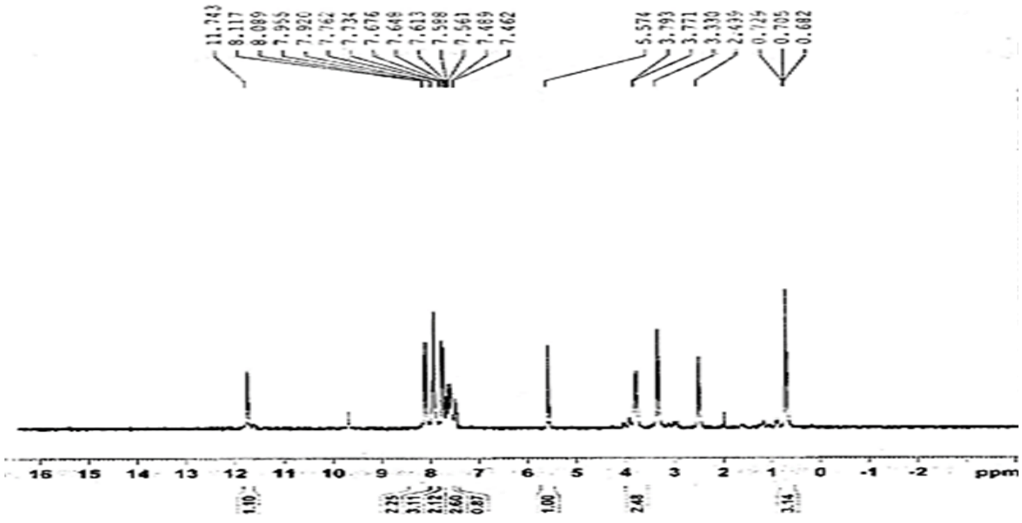

Fig. S10. ^{1}H NMR spectrum of compound **4d**.

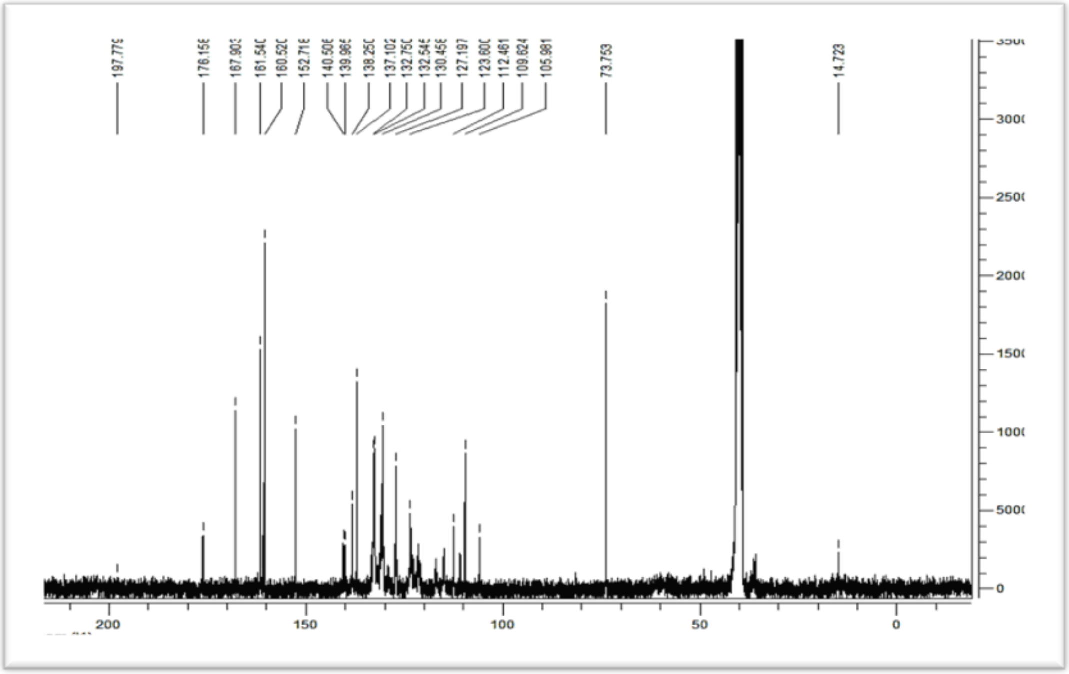

Fig. S11. ^{13}C NMR spectrum of compound **4d**.

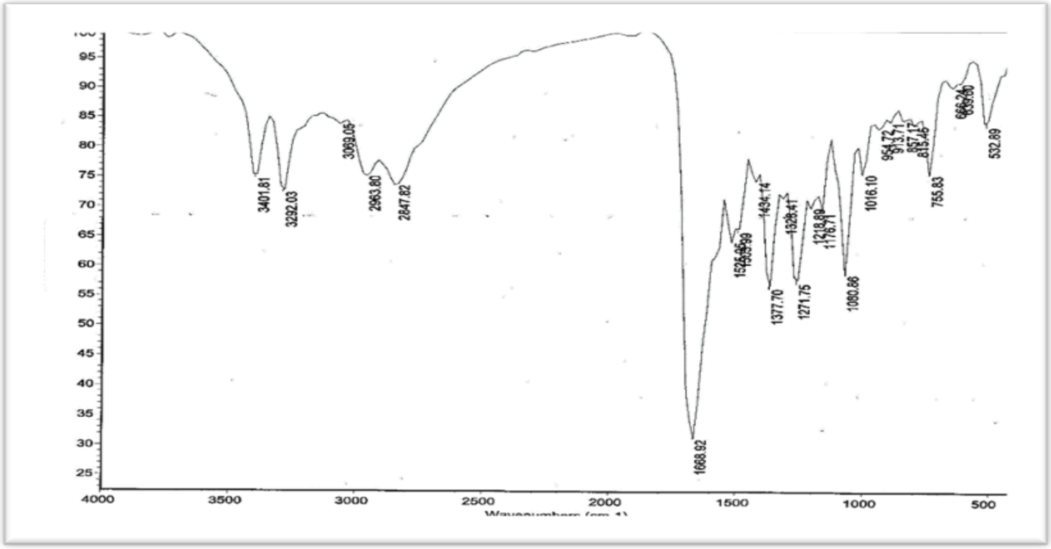

Fig. S12. IR spectrum of compound **4d**.

Ethyl 2-amino-4-(4-methylbenzoyl)-5-oxo-5,6-dihydro-4*H*-pyrano[3,2-*c*]quinoline-3-carboxylate (4d)

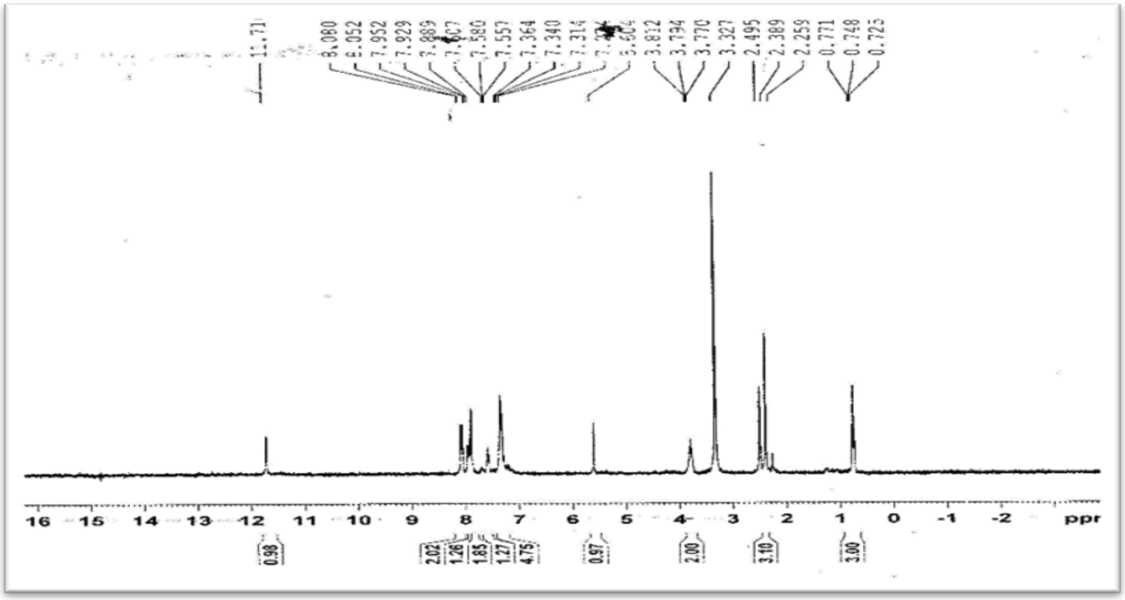

Fig. S13. ^{1}H NMR spectrum of compound **4e**.

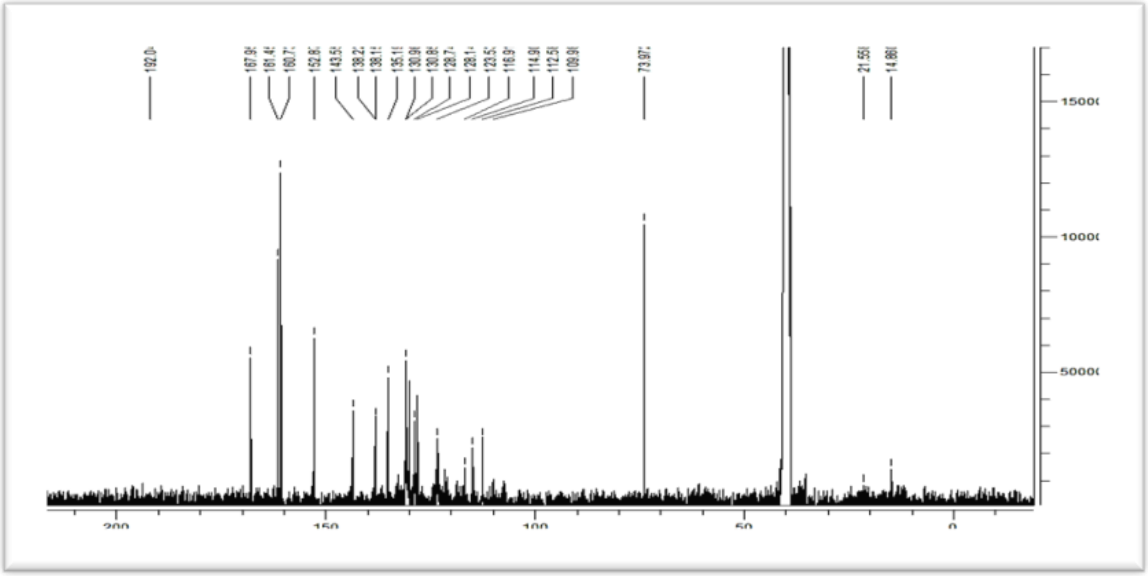

Fig. S14. ^{13}C NMR spectrum of compound **4e**.

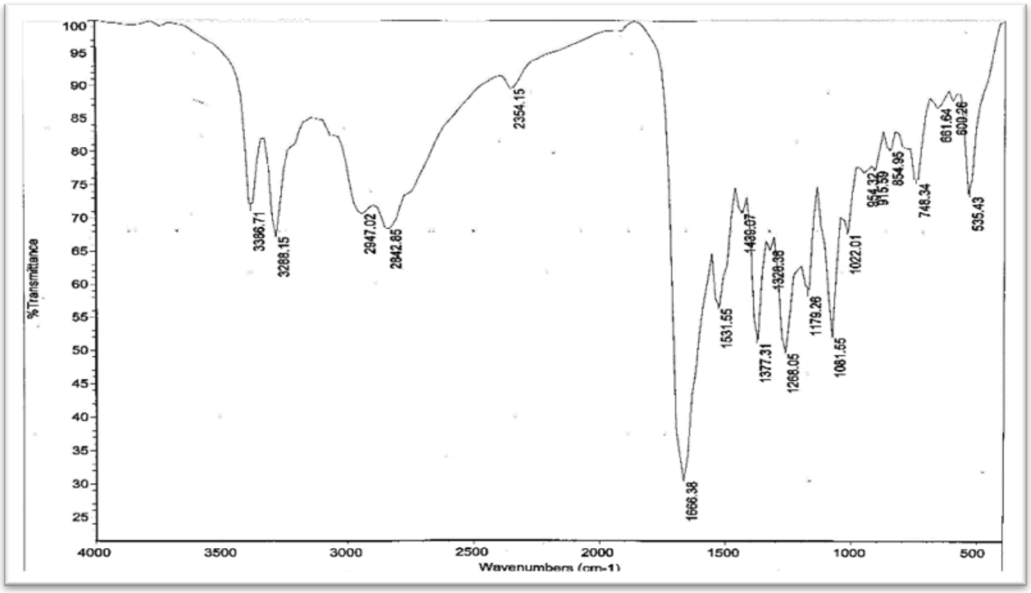

Fig. S15. IR spectrum of compound 4e.

Ethyl 2-amino-4-(4-fluorobenzoyl)-5-oxo-5,6-dihydro-4*H*-pyrano[3,2-*c*]quinoline-3-carboxylate (4f)

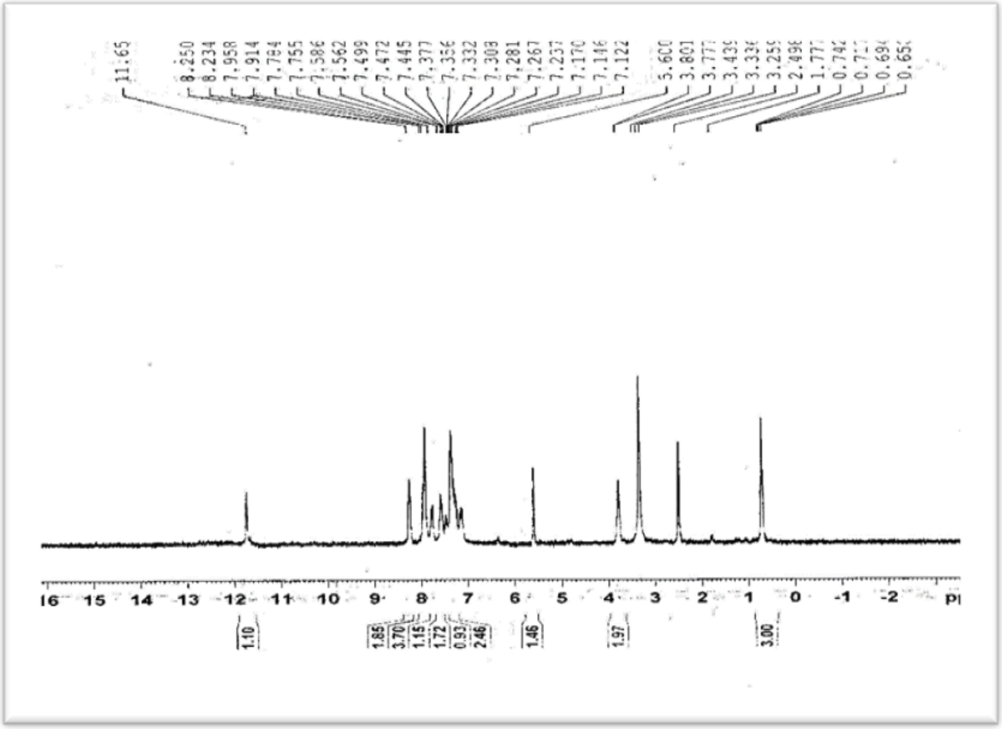

Fig. S16. ^{1}H NMR spectrum of compound **4f**.

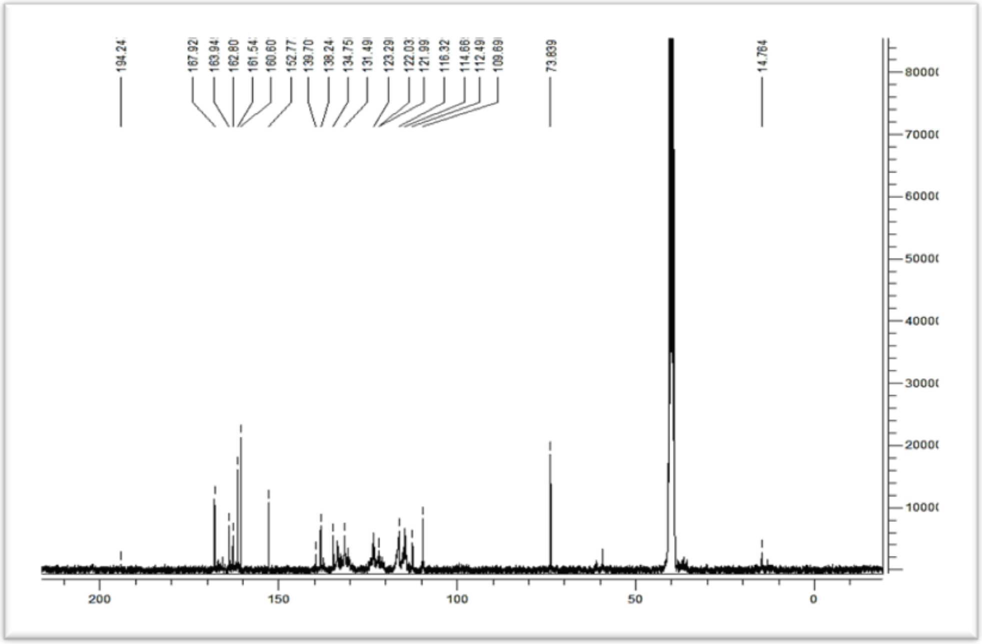

Fig. S17. ^{13}C NMR spectrum of compound **4f**.

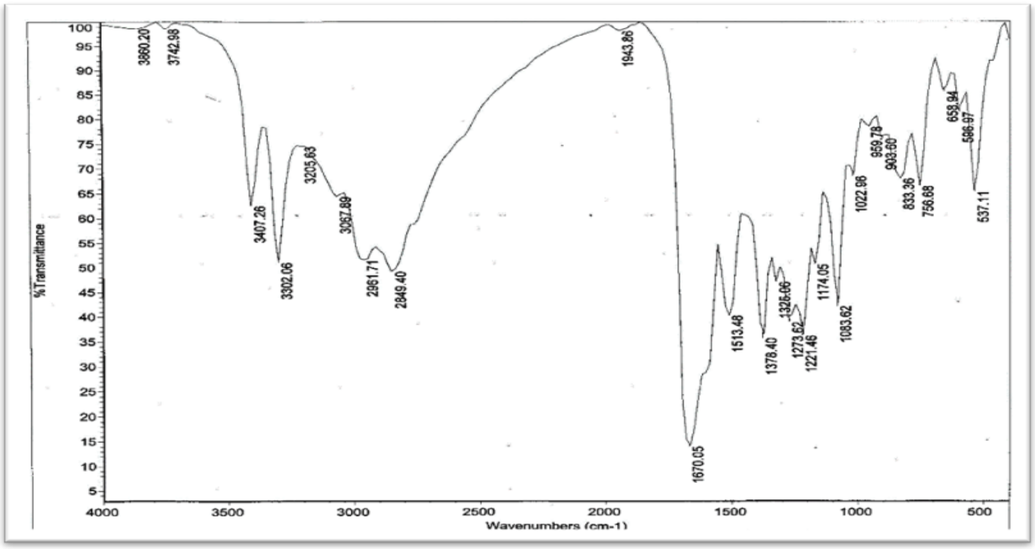

Fig. S18. IR spectrum of compound **4f**.

Ethyl 2-amino-4-(3-methoxybenzoyl)-5-oxo-5,6-dihydro-4*H*-pyrano[3,2-*c*]quinoline-3-carboxylate (4g)

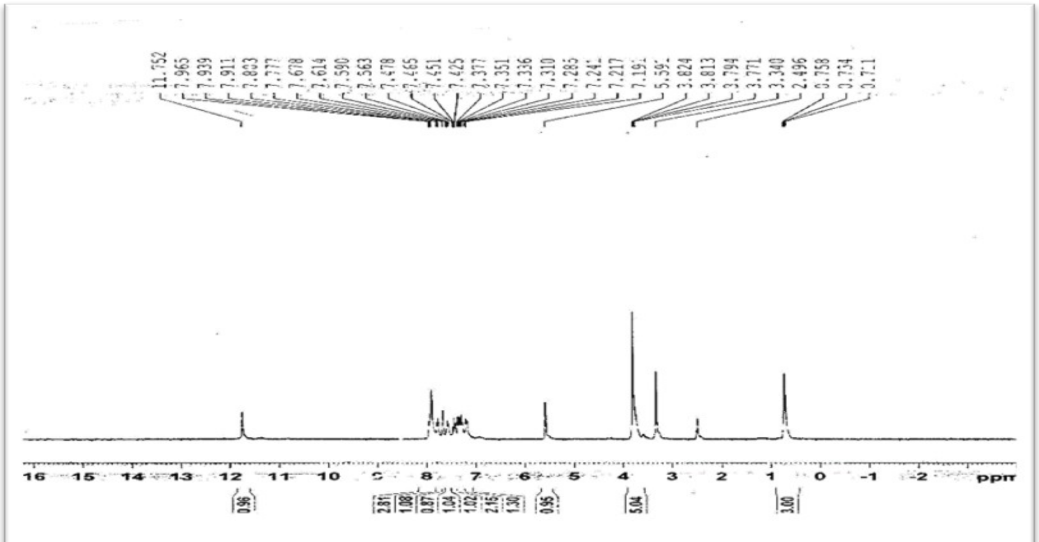

Fig. S19. ^{1}H NMR spectrum of compound **4g**.

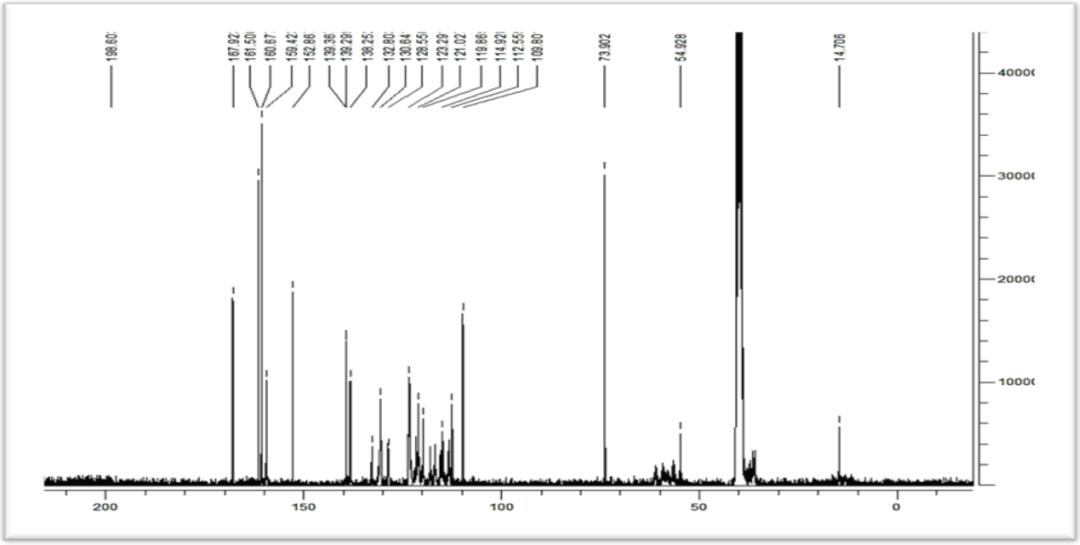

Fig. S20. ^{13}C NMR spectrum of compound **4g**.

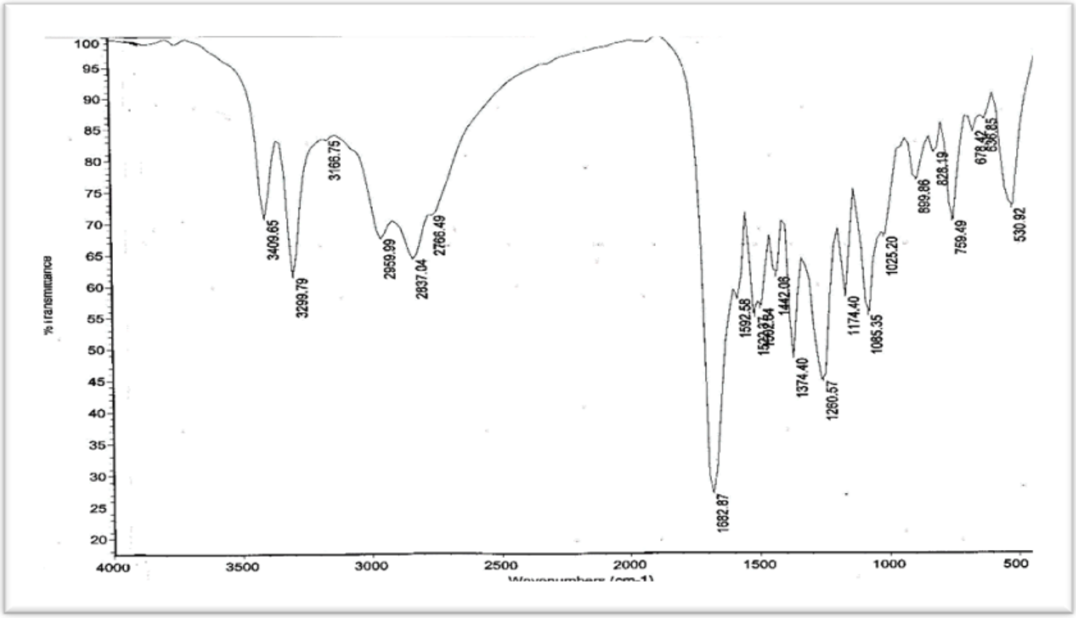

Fig. S21. IR spectrum of compound **4g**.

Ethyl 2-amino-4-(4-nitro)-5-oxo-5,6-dihydro-4*H*-pyrano[3,2-*c*]quinoline-3-carboxylate (4h)

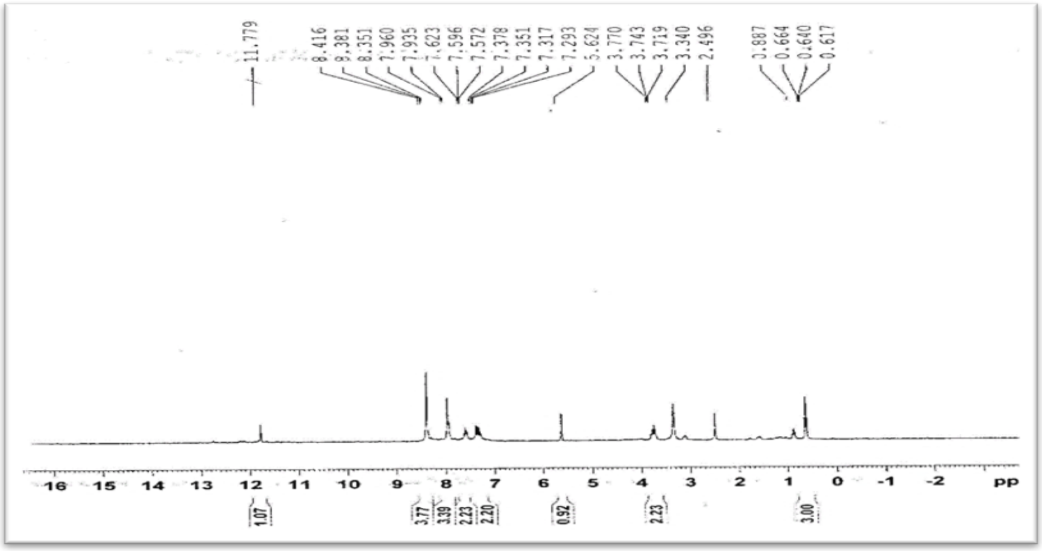

Fig. S22. ^{1}H NMR spectrum of compound 4h.

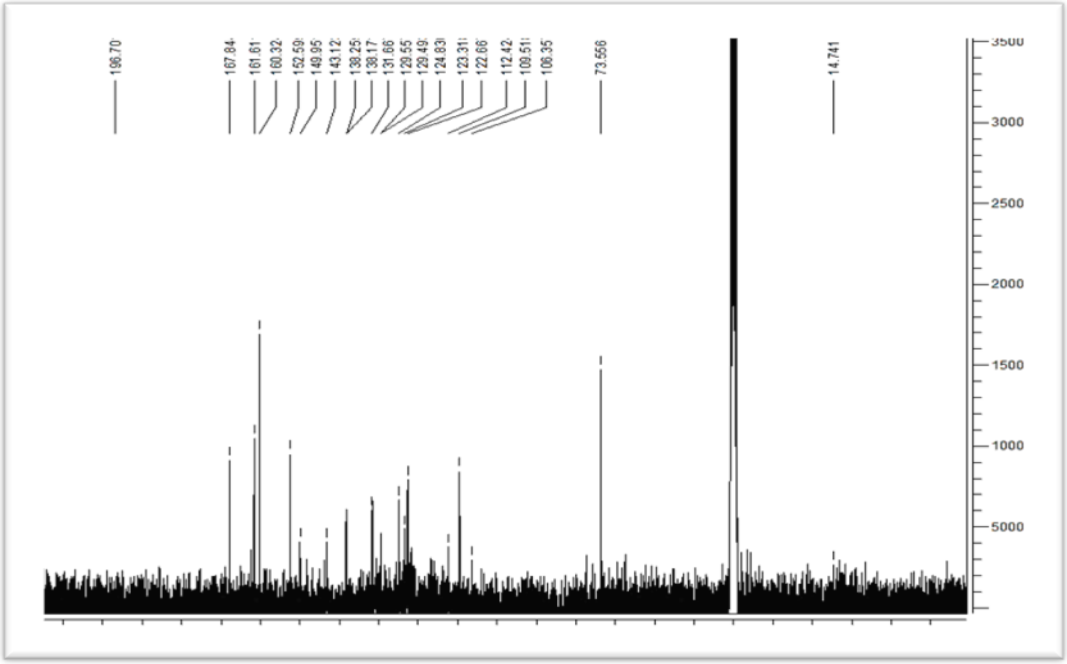

Fig. S23. ^{13}C NMR spectrum of compound **4h**.

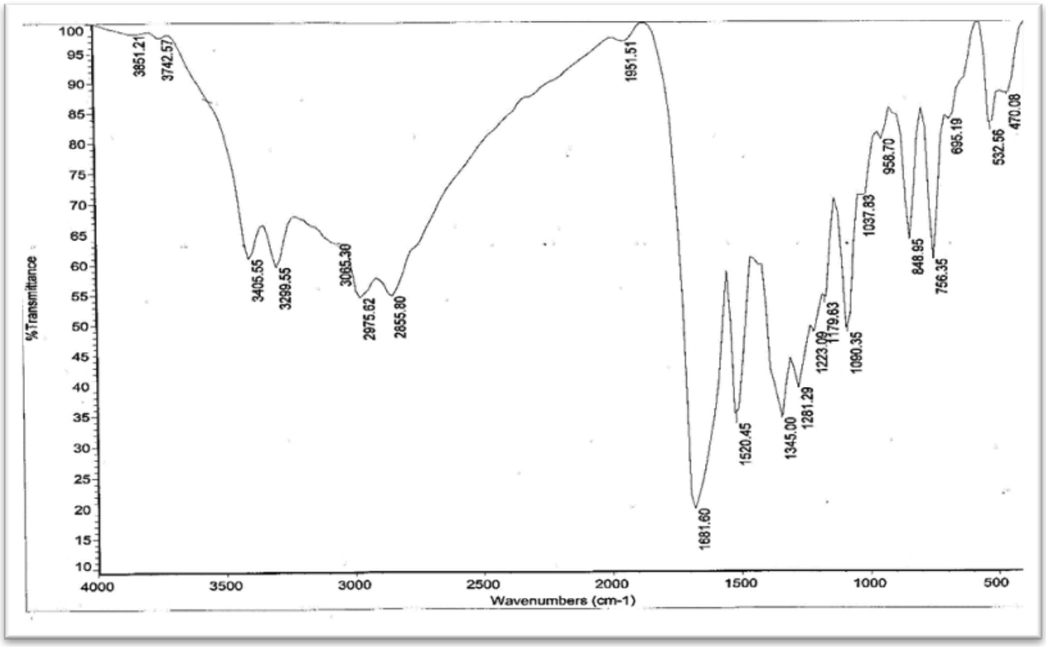

Fig. S24. IR spectrum of compound **4h**.

References

[S1] Hummers, Jr., William, S. & Richard, E. Offeman. Preparation of graphitic oxide. *J. Am. Chem. Soc.* **80**, 1339–1339 (1958).

[S2] Rostamnia, S., et al. Surfactant-exfoliated highly dispersive Pd-supported graphene oxide nanocomposite as a catalyst for aerobic aqueous oxidations of alcohols. *ChemCatChem* **7**, 1678–1683 (2015).

[S3] Qilin, L., et al. Antimicrobial nanomaterials for water disinfection and microbial control: potential applications and implications. *Water Research* **42,** 4591–4602 (2008).

[S4] Poursattar Marjani, A., Khalafy, J. & Farajollahi, A. Synthesis of ethyl 2-amino-4-benzoyl-5-oxo-5,6-dihydro-4*H*-pyrano[3,2-*c*]quinoline-3-carboxylates by a one-pot, three-component reaction in the presence of TPAB. *Journal of Heterocyclic Chemistry* **56**, 268–274 (2019).
